# Overexpression of an endogenous type 2 diacylglycerol acyltransferase in the marine diatom *Phaeodactylum tricornutum* enhances lipid production and omega-3 long-chain polyunsaturated fatty acid content

**DOI:** 10.1186/s13068-020-01726-8

**Published:** 2020-05-14

**Authors:** Richard P. Haslam, Mary L. Hamilton, Chloe K. Economou, Richard Smith, Kirsty L. Hassall, Johnathan A. Napier, Olga Sayanova

**Affiliations:** 1 Department of Plant Sciences, Rothamsted Research, Harpenden, Herts AL5 2JQ UK; 2 St Albans Girls School, St Albans, Hertfordshire, AL3 6DB UK; 3 School of Biological and Chemical Sciences, Queen Mary University of London, Mile End Road, London, E1 4NS UK; 4 Algenuity, Eden Laboratory, Broadmead Road, Stewartby, BEDS, Bedford, MK43 9ND UK; 5 Department of Computational and Analytical Sciences, Rothamsted Research, Harpenden, Herts AL5 2JQ UK

**Keywords:** Acyl-CoA:diacylglycerol acyltransferase (DGAT), Eicosapentaenoic acid, Docosahexaenoic acid, Triacylgycerol, Omega-3 fatty acids, *Phaeodactylum tricornutum*

## Abstract

**Background:**

Oleaginous microalgae represent a valuable resource for the production of high-value molecules. Considering the importance of omega-3 long-chain polyunsaturated fatty acids (LC-PUFAs) for human health and nutrition the yields of high-value eicosapentaenoic acid (EPA) and docosahexaenoic acid (DHA) require significant improvement to meet demand; however, the current cost of production remains high. A promising approach is to metabolically engineer strains with enhanced levels of triacylglycerols (TAGs) enriched in EPA and DHA.

**Results:**

Recently, we have engineered the marine diatom *Phaeodactylum tricornutum* to accumulate enhanced levels of DHA in TAG. To further improve the incorporation of omega-3 LC-PUFAs in TAG, we focused our effort on the identification of a type 2 acyl-CoA:diacylglycerol acyltransferase (DGAT) capable of improving lipid production and the incorporation of DHA in TAG. DGAT is a key enzyme in lipid synthesis. Following a diatom based in vivo screen of candidate DGATs, a native *P. tricornutum DGAT2B* was taken forward for detailed characterisation. Overexpression of the endogenous *P. tricornutum DGAT2B* was confirmed by qRT-PCR and the transgenic strain grew successfully in comparison to wildtype. *PtDGAT2B* has broad substrate specificity with preferences for C16 and LC-PUFAs acyl groups. Moreover, the overexpression of an endogenous *DGAT2B* resulted in higher lipid yields and enhanced levels of DHA in TAG. Furthermore, a combined overexpression of the endogenous *DGAT2B* and ectopic expression of a Δ5-elongase showed how iterative metabolic engineering can be used to increase DHA and TAG content, irrespective of nitrogen treatment.

**Conclusion:**

This study provides further insight into lipid metabolism in *P. tricornutum* and suggests a metabolic engineering approach for the efficient production of EPA and DHA in microalgae.

## Background

Microalgal strains with a high-lipid content, stored as triacylglycerol (TAG), are considered a valuable food source and a promising feedstock to produce high-value molecules [1]. As a result, research into the pathways that regulate TAG composition and accumulation have received much attention ([2, 3] and references therein). Often microalgae accumulate high quantities of TAGs in response to abiotic stress, e.g. nutrient starvation, high temperature, salinity, pH or light intensity [4]. The application of such environmental stresses can be an effective strategy for increasing lipid production in microalgae; however, their negative effect on cell growth is a major bottleneck for reducing production costs. Therefore, new efficient strategies are needed to increase economic viability of production [5]. A promising approach is the use of genetic engineering for generating improved strains with desirable characteristics such as fatty acid (FA) composition, enhanced lipid biosynthesis and high growth rates.

Within the complex network of activities associated with TAG assembly, acyl-CoA:diacylglycerol acyltransferases (DGATs) and phospholipid:diacylglcerol acyltransferases (PDATs) have been shown to play an important role in TAG accumulation (for reviews see [1] and [6]). DGAT (EC 2.3.1.20) catalyses the final committed reaction in the acyl-CoA dependent Kennedy TAG biosynthetic pathway by the esterification of acyl-CoA to a diacylglycerol (DAG) moiety [1, 7]. At least three classes of DGATs have been identified in eukaryotes. Of these, two types, designated DGAT1 and DGAT2, are membrane-bound acyltransferases and play a major role in TAG biosynthesis. Whereas DGAT3 is a soluble isoform characterised in peanut (*Arachis hypogaea*) and *Arabidopsis thaliana*, where it is thought to affect the flux between the cytosolic acyl-CoA pool and TAG [1, 8, 9]. The pivotal role of DGAT in TAG synthesis has been demonstrated by the increase in seed oil content and altered FA composition resulting from the ectopic expression of plant DGAT1 and DGAT2s (for Refs see [1, 10, 11]). Therefore, one beneficial route for the metabolic engineering of microalgae for increased oil content and tailored FA composition may be via the manipulation of microalgal DGAT gene expression.

Most eukaryotic organisms contain at least one copy from the DGAT family of three classes [1]. To date, genome sequencing of photosynthetic microalgae has revealed the presence of a single gene copy encoding the type 1 DGAT, apart from *Nannochloropsis oceanica* IMET1, where two isoforms were identified [12]. By contrast, multiple copies of DGAT2 genes have been annotated in microalgal genomes, for example five DGAT2s have been found in *Phaeodactylum tricornutum* and 12 copies in *N. oceanica* [13, 14]. Despite this diversity, currently only a few microalgal DGATs have been functionally characterised. In most instances, attempts to functionally characterise DGAT isoforms have used a *Saccharomyces cerevisiae* mutant strain (H1246) lacking endogenous DGAT and PDAT genes. Using this approach DGAT1 isoform from *P. tricornutum* was successfully characterised, demonstrating a preference to produce TAGs with a high level of saturated fatty acids (16:0 and 18:0) [15]. Numerous DGAT2 genes have been putatively identified in microalgae, e.g. *P. tricornutum, N. oceanica*, *Chlamydomonas reinhardtii and Ostreococcus tauri,* for subsequent characterisation in TAG-deficient *S. cerevisiae* [13, 16–19]. Not all the genes tested restored the TAG phenotype, but of those that did, many showed differing substrate specificity for mono- and polyunsaturated acyl-CoA substrates, e.g. *O. tauri DGAT2B* showed broad substrate specificity, accepting saturated as well as mono- and polyunsaturated acyl-CoAs as substrates [19], whilst DGAT3 isoform from *P. tricornutum* (*PtDGAT3*) displayed a preference for the incorporation of unsaturated C18-fatty acids [20].

In *P. tricornutum* four putative type 2 DGATs, annotated as *PtDGAT2A* (Phatr2_49462), *PtDGAT2B* (Phatr2_49544), *PtDGAT2C* (Phatr2_31662) and *PtDGAT2D* (Phatr2_43469), have been characterised in TAG-deficient yeast [19]. Of these only *PtDGAT2B* restored lipid body formation and showed a preferential substrate specificity for unsaturated FAs. In addition, transcript analysis revealed that *PtDGAT2A* and *PtDGAT2B* were upregulated prior to the onset of TAG accumulation. Further studies indicated that the overexpression of *PtDGAT2D* in *P. tricornutum* resulted in an increased flux of photosynthetically fixed carbon towards lipids leading to a twofold higher lipid content in transgenic cells compared to wild type (WT), alongside a reduction (15%) in cell growth [21]. In contrast, overexpression of a chloroplast *DGAT2* homologue from the diatom *T. pseudonana* (Thaps3_25595) significantly increased TAG accumulation without a negative effect on growth or biomass accumulation [22].

Collectively, these studies illustrate how manipulation of genes encoding DGATin microalgae can increase their lipid content and modify fatty acid composition. Moreover, it is reasonable to suggest that they might participate in producing TAGs with enhanced levels of long-chain polyunsaturated fatty acids (LC-PUFAs) in microalgae (i.e. increasing incorporation of eicosapentaenoic acid, 20:5Δ^5,8,11,14,17^, EPA and docosahexaenoic acid, 22:6Δ^4,7,10,13,16,19^, DHA). Recently, we have engineered *P. tricornutum* to accumulate enhanced levels of DHA by overexpressing the Δ5-elongase from the picoalga *O. tauri* [23] and demonstrated the potential of this transgenic strain for industrial production of omega-3 LC-PUFAs in phototrophic and heterotrophic conditions [24, 25]. The DHA content in transgenic Pt_OtElo5 was increased up to eightfold that of WT levels and several novel LC-PUFA containing TAG species were detected. Further work has demonstrated the feasibility of multifunctionalising the Pt_OtElo5 strain, combining the accumulation of high levels of EPA and DHA with recombinant protein production [26]. In the work presented here, we studied the effect of transgenic expression of the three most promising candidate *DGAT2s*, namely *P. tricornutum DGAT2A*, *DGAT2B* and *T. pseudonana DGAT2* (TpDGAT2), on lipid accumulation and omega-3 LC-PUFA incorporation into TAG in WT and transgenic strains. The impact of these enzymes on lipid production in vivo was studied via lipidomic analysis. Our results demonstrate that overexpression of *PtDGAT2B* significantly enhances TAG production and alters FA composition during nitrogen-replete and nitrogen-limiting conditions. This study provides better insight into TAG biosynthesis in *P. tricornutum* and suggests a promising approach for tailoring oil composition in microalgae.

## Results

### Generation of transgenic *P. tricornutum* strains overexpressing candidate DGAT2s

**Fig. 1:**
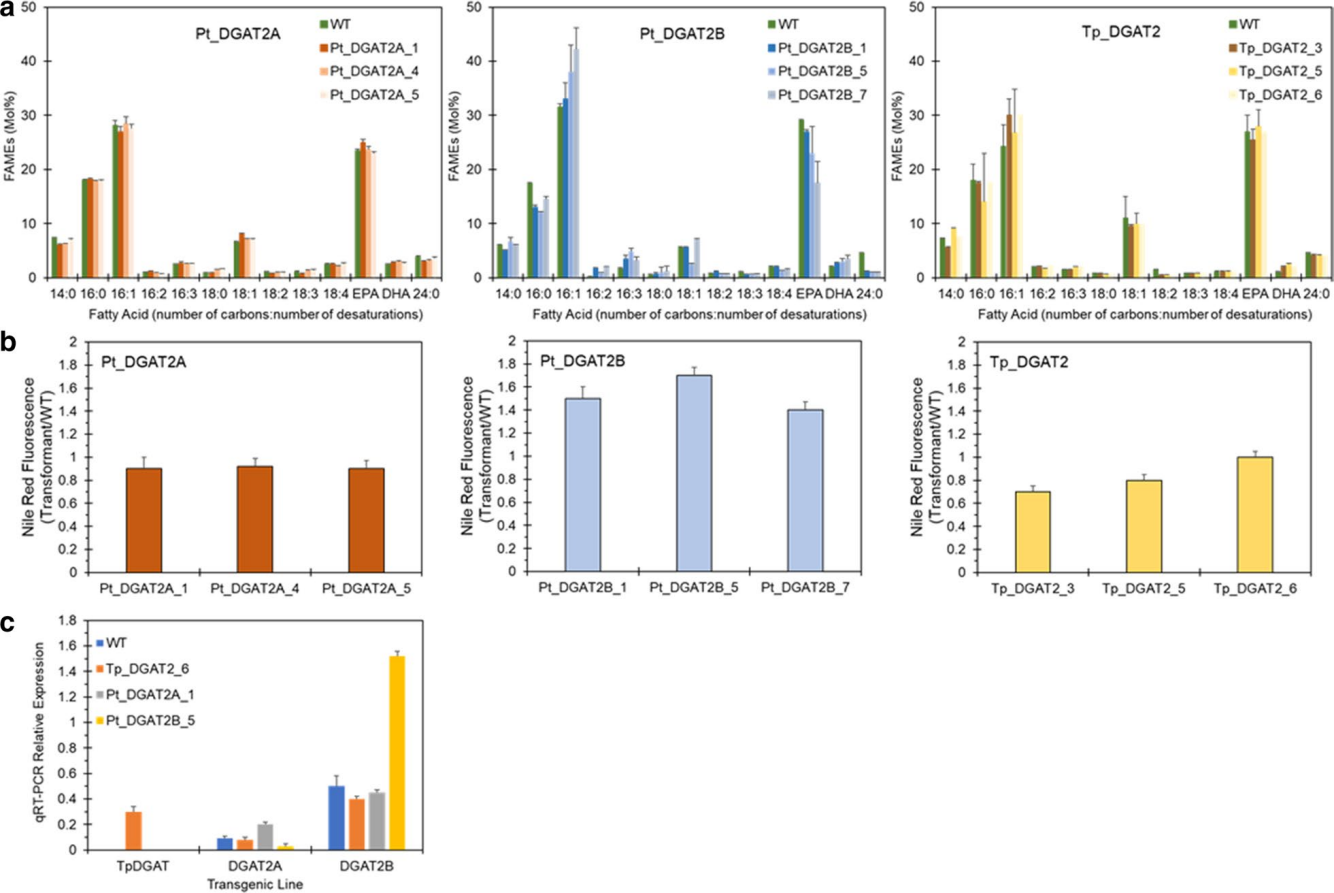
Overexpression of DGAT2 isoforms in *P. tricornutum*. **a** Fatty acid composition (Mol%) of independent transgenic lines overexpressing DGAT2 genes during S phase. Each measurement is the average of three technical replicas (± standard error). **b** Neutral lipid content of transgenic cells overexpressing different DGAT2 isoforms assessed by Nile Red fluorescence. Each data point represents an average of three biological replicas. **c** Quantitative RT-PCR analysis of the transcript levels of DGAT2 isoforms. Transgenic algal cultures overexpressing *TpDGAT2*, *PtDGAT2A* and *PtDGAT2B* were grown under continuous light for 3 days in standard F/2 medium. Relative expression levels were normalised to that of the housekeeping gene *RPS*

The native codon sequences for *P. tricornutum DGAT2A*, *DGAT2B* and a *T. pseudonana DGAT2* (*TpDGAT2*), codon-optimised for expression in *P. tricornutum*, were cloned into the pPhOS2 vector [23] and the resulting constructs (Pt_DGAT2A, Pt_DGAT2B and Pt_TpDGAT2) were then transformed into *P. tricornutum* via biolistics (as described in [23]). Multiple zeocin-resistant colonies (> 10) obtained for each construct were confirmed by PCR and then used to inoculate cultures for further screening by gas chromatography coupled to ion flame detection (GC-FID) analysis of fatty acid methyl esters (FAMEs). From this screening, we obtained multiple independent transgenic lines for each construct (Additional file 1: Table S1). The FA profiles and lipid content of selected independent overexpression lines were then analysed and compared to that of WT. As previously observed [23], the main FAs in total lipid extracts of WT and transgenic cells in decreasing order were C16:1, EPA, C16:0 and C14:0 (Fig. 1a). Transgenic lines overexpressing *Pt_DGAT2B* and *Tp_DGAT2* demonstrated slightly elevated DHA levels.

To visualise the impact of transgene expression, a Nile Red fluorescence assay was used to evaluate the ability of the candidate DGAT2 overexpressing lines to accumulate enhanced levels of neutral lipids. Previously, analysis of transcript levels of the DGAT2 genes under replete growth conditions showed that the expression level of *PtDGAT2A* and *PtDGAT2B* progressively increased from day 2 to day 4, peaked and then decreased to a low level in the following days [13]. Hence, transgenic strains overexpressing DGAT2s were cultivated for 3 days in replete F/2 medium to the early exponential (E) phase (2.5 × 10^6^ cells). Nile Red staining of neutral lipids showed substantial differences between different DGAT2 clones and WT (Fig. 1b). All *PtDGAT2B* expressing clones showed a marked increase of TAG, whilst Pt_DGAT2A and Tp_DGAT2 cells did not accumulate oil levels compared to WT cells.

To confirm gene transcription and elevated expression of DGAT2 genes, quantitative real-time PCR (qRT-PCR) was carried out on selected transgenic lines. Based on the results of Nile Red staining, one of the most promising transgenic lines overexpressing each of the DGAT2 isoforms were characterised. Transgenic cultures were grown in F/2 medium and RNA extracted at the early E phase. Subsequent qRT-PCR analysis confirmed expression of *Pt_DGAT2A_1*, *Pt_DGAT2B_5* and *Tp_DGAT2_6* transcripts in the selected transgenic strains and demonstrated that the *Pt_DGAT2B* transgene is expressed at higher levels than the other two transgenes, *Tp_DGAT2* and *Pt_DGAT2A* (Fig. 1c). Although the genes were under the control of the same promoter, the relative expression levels for the three DGAT2 isoforms were significantly different (*p* < 0.001, for expression of both *DGAT2A* and of *DGAT2B* genes). For cell lines overexpressing native *PtDGAT2A* and *PtDGAT2B*, transcripts were increased relative to WT by 2.3- and 3.0-fold, respectively. Based on the combined analysis of FA profiles, Nile Red staining and qRT-PCR analysis, the clone with the highest lipid content, Pt_DGAT2B_5, now designated DGAT2B, was taken forward for further detailed analysis.

### Generation of transgenic *P. tricornutum* strains coexpressing *PtDGAT2B* and Δ5-elongase from the picoalga *O. tauri*

**Fig. 2:**
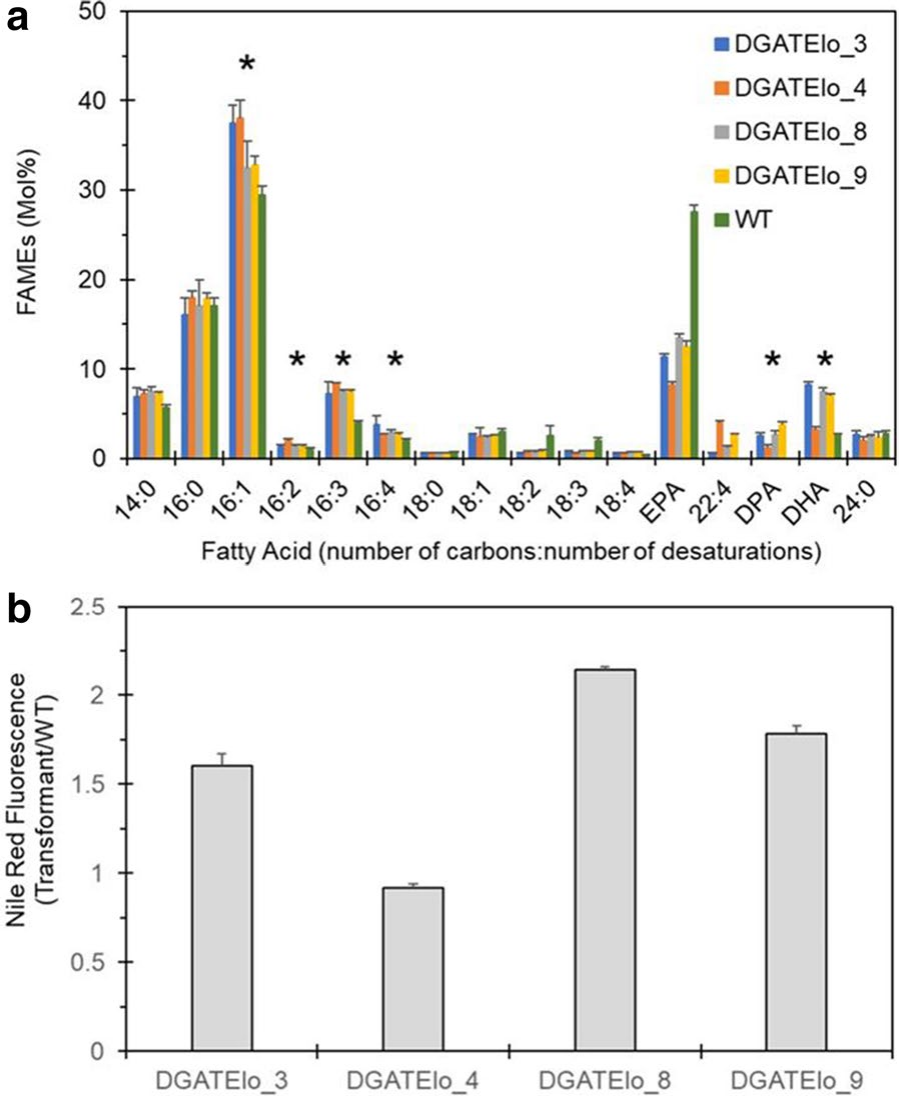
Functional characterisation of transgenic *P. tricornutum* lines coexpressing *DGAT2B* and *OtElo5*. **a** Fatty acid composition of WT and DGATElo5 cells in the S phase of growth in F/2 media. Each measurement is the average of three biological replicas (± standard error). **b** Neutral lipid content of DGATElo transgenic clones assessed by Nile Red fluorescence (refer to “Methods” ***section for experimental description). Relative level of fluorescence in the transformants compared to WT cells. Values are the average of three replicated experiments. Error bars indicate standard error. Fatty acids which are more abundant in transgenic cells compared to WT are indicated by an asterisk (*)

To evaluate the effect of *PtDGAT2B* overexpression on the incorporation of omega-3 LC-PUFAs in TAG, coding sequences for the *O. tauri* Δ5-elongase (*OtElo5*) and *PtDGAT2B* were cloned in the previously described pPhOS2 vector [23], generating DGATElo construct. Ten individual zeocin-resistant clones were confirmed by PCR and screened for FA content (Additional file 2: Table S2). Four of the independent clones containing the highest levels of the new fatty acid, docosapentaenoic acid (DPA, 22:5n-3), the product of elongating activity of Δ5-elongase, and DHA were selected for further analysis. FAMEs analysis of the selected transgenic strains confirmed the presence of DPA in the range of 1.8–4.6% accompanied by increased DHA levels (up to 3.8–8.5% compared to 1.8% in WT) in the stationary (S) phase of growth (Fig. 2a). Compared to WT, transgenic clones demonstrated substantially altered fatty acid profiles, containing on average twofold higher levels of 16:3 and 16:4, whilst the levels of C18:2 and C18:3n-3 were slightly reduced. The rise in 16:3 and 16:4 unsaturated FA is most likely due to the increase in substrate levels (C16:1) available for further desaturation by stroma desaturases. The lipid content of these clones was further screened by Nile Red assay (Fig. 2b). The clone with the highest level of DHA and enhanced neutral lipid content, DGATElo_8 (designated DGATElo), was taken forward for further analysis.

### Cell growth and transgene expression: the impact of *PtDGAT2B* overexpression under N-replete and N-deplete conditions

**Fig. 3:**
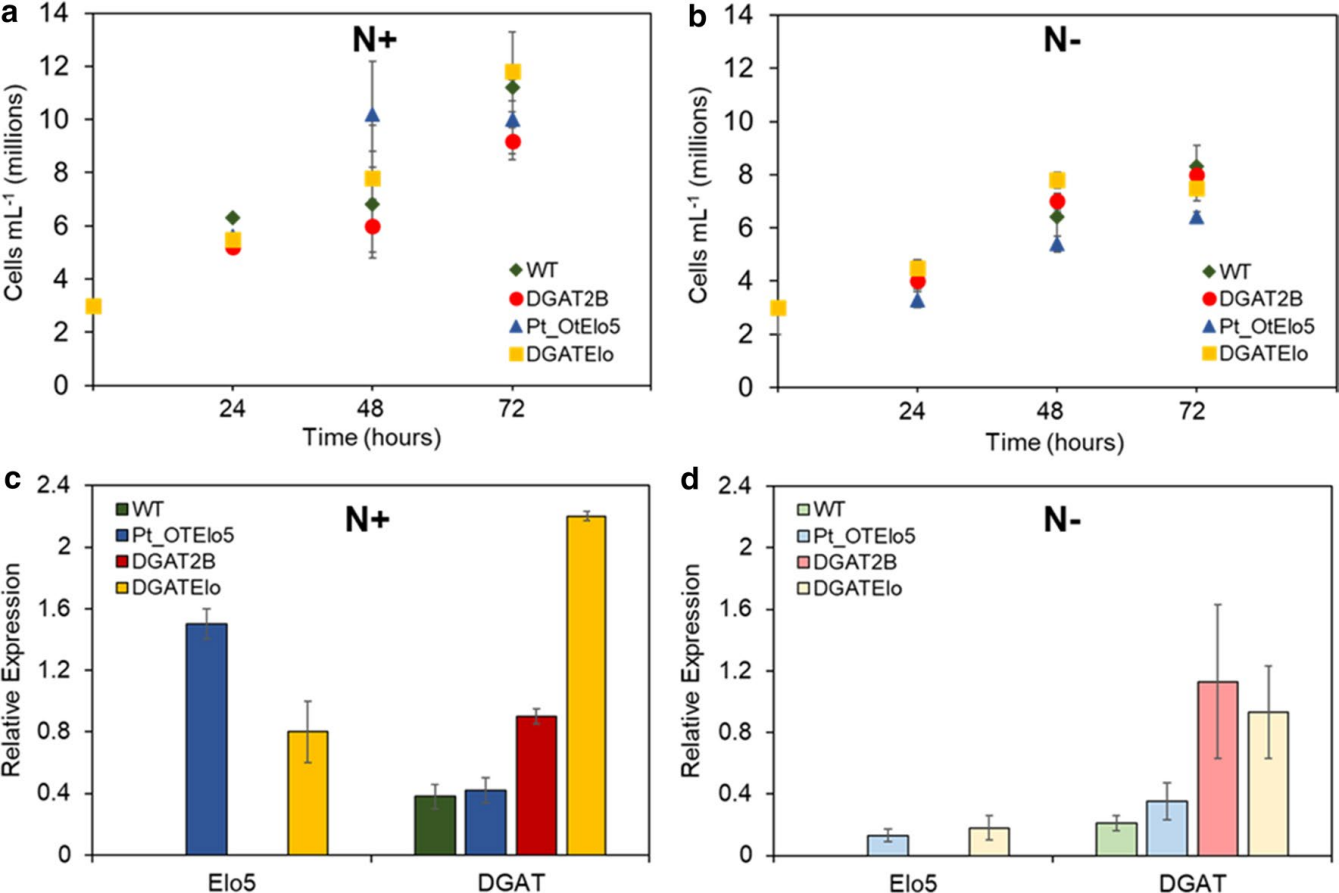
Cell growth and relative expression of transgenes in WT and transgenic *P. tricornutum* strains. **a**, **b** Cellular growth of WT and transgenic Pt_OtElo5, DGAT2B and DGATElo strains. Cells grown under N+ and N− conditions were harvested for lipid analysis where indicated. Values are the average of four experiments (± standard error). **c**, **d** The relative expression of *OtElo5* and *DGAT2B* transcript levels analysed by qRT-PCR in transformants growing under N + and N− conditions. Algal cultures were grown under continuous light for 72 h in standard F/2 medium containing nitrogen (N +) or in N-deprived (N−) conditions. Relative transcript abundance was normalised to that of housekeeping gene *RPS* and calculated using the 2^−∆∆CT^ method. Values are the average of three replicated experiments. Error bars indicate standard error

Nutrient deprivation has been shown to induce TAG accumulation in microalgae [27]. To address the role of *PtDGAT2B* in TAG synthesis, we studied cell growth under N-replete and N-deplete conditions during the most active phase of oil accumulation (72 h). N-replete F/2 media was supplemented with additional P and N according to Abida et al. to ensure that these elements were not limiting during cell growth [28]. N-deplete media contained F/2 enriched with added P and no N. The growth rates and lipid accumulation of transgenic lines were determined under both N-replete and N-depleted conditions at 24-h intervals for a total of 72 h. The analysis of the growth profiles showed that there were no differences in growth patterns between the transgenic lines and the WT under both conditions. The rate of cell division under both N-replete and N-deplete conditions was both linear and consistent for transgenic and WT cells (Fig. 3a, b). Whereas the overall level of cell division differed substantially between the two nitrogen conditions (p < 0.01), decreasing in the N-deprived cultures. To confirm transcription of both the *OtElo5* and *PtDGAT2B* genes during the experiment, RNA was extracted from triplicate cultures at 72 h of growth after resuspension in either N-replete or N-free media. Following cDNA synthesis qRT-PCR confirmed that overexpression of *PtDGAT2B* and transcription of the *OtElo5* gene were occurring under experimental conditions (Fig. 3c, d). We have previously described the successful expression of *OtElo5* under the endogenous *fcpA* promoter of highly expressed *fcpA* gene [23]. However, *fcp* promoters may not be strong enough to overexpress introduced activities due to the presence of light-responsive *cis*-regulatory elements [29] and stability under low-nutrient conditions. Seo et al. showed that the endogenous elongation factor 2 (EF2) promoter isolated from *P. tricornutum* drove the expression of a transgene 1.2-fold stronger than that driven by the *fcp* promoter in light conditions and was stable throughout light and dark cycles [30]. However, recently we have shown that the expression of the Δ5-elongase (*OtElo5*) gene with an EF2 promoter resulted in comparable levels of EPA and DHA to that in transgenic strains in which the *OtElo5* gene was under control of the *fcpA* promoter [26]. In this study, we tested both promotors’ efficacies in response to Nitrogen treatment in transgenic lines expressing *OtElo5* (Additional file 3: Table S3). The results indicate that the *fcpA* promoter is suitable for transgene expression of *DGAT2B* in *P. tricornutum* when grown in different N conditions.

### Effect of *PtDGAT2B* overexpression on fatty acid composition under N-replete and N-deplete conditions

**Fig. 4:**
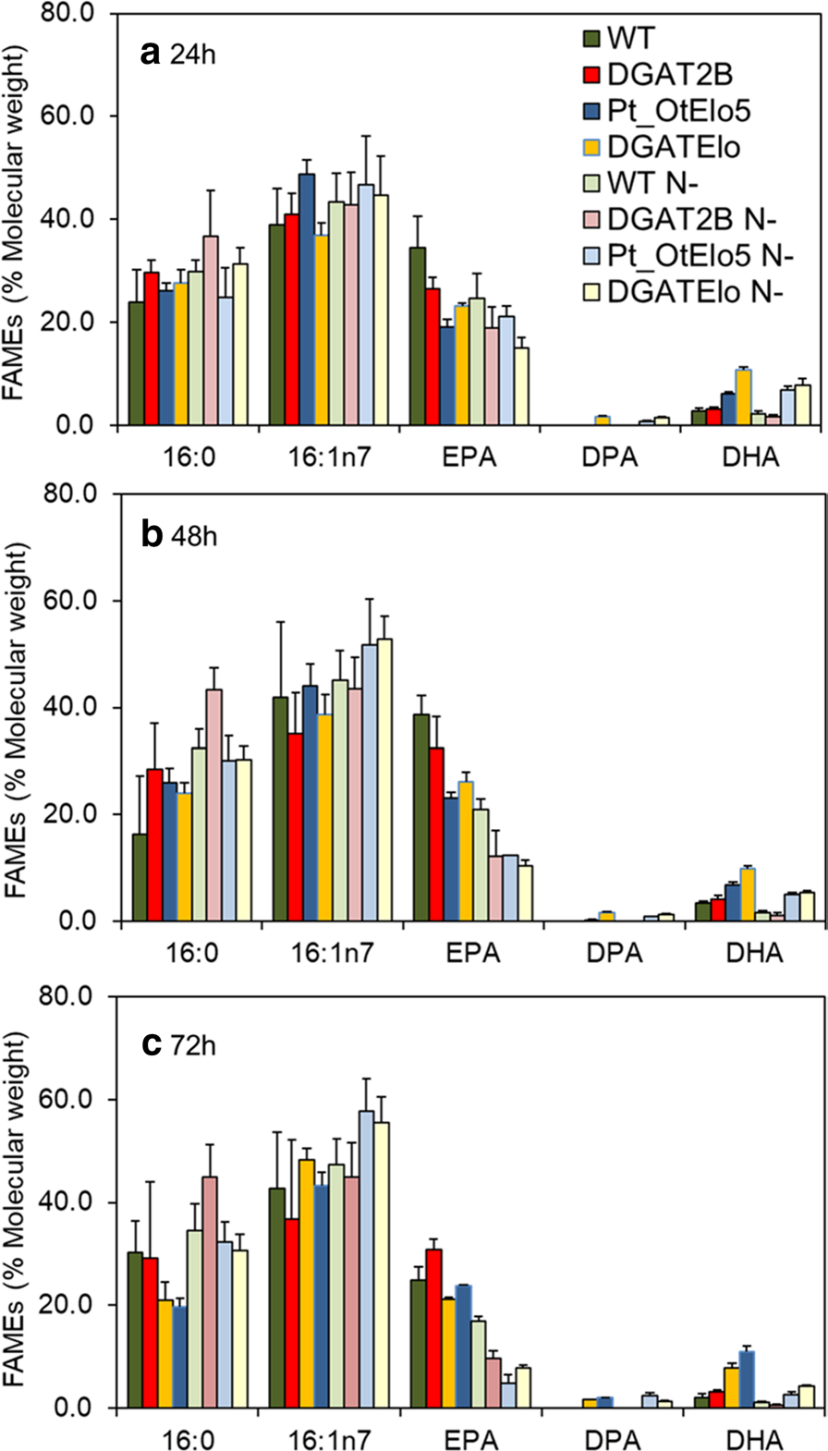
Fatty acid composition (Mol  %) of WT and transgenic (Pt_OtElo5, DGAT2B and DGATElo) *P. tricornutum.* Cells cultivated in N-replete (N + , dark bars) and N-deplete (N−, pale bars) medium at 24 (**a**), 48 (**b**) and 72 (**c**) hours. Each measurement is the average of minimum four technical replicates. Error bars indicate standard error

To assess the impact of *PtDGAT2B* heterologous expression on cell FA profiles, cells were grown in N-replete and N-deplete conditions and analysed at 24, 48 and 72 h time points, respectively. The fatty acid profiles of selected independent overexpression lines DGAT2B and DGATElo were then compared to that of WT and transgenic Pt_OtElo5. Fatty acid composition (Mol%) was clearly affected by N treatment, for example DGAT2B cells accumulated slightly higher levels of DHA in comparison to WT in N-replete conditions (Fig. 4). Irrespective of N treatment, the highest DHA levels were observed in DGATElo cells. As expected, cells expressing *OtElo5* (Pt_OtElo5 and DGATElo) contained decreased EPA compared to that of DGAT2B and WT at all time points due to the action of the Δ5-elongase (Fig. 4). The novel FA DPA was only present in the strains expressing *OtElo5* (Pt_OtElo5 and DGATElo).

When total FAMEs were quantified (expressed as nmol mg^−1^), the main differences between strains were observed in the content of 16:0 and 16:1 (Additional file 4: Figure S1). As expected, [28, 31], N deprivation was accompanied by significant changes in the levels of 16:0 and 16:1 (*P* < 0.001) and time (*P* < 0.001). The abundance of 16:0 and 16:1 was higher in N-starved cultures and increased over time (*P* = 0.002 and *P* = 0.019, respectively) in all strains. Higher levels of 16:0 and 16:1 were observed in cells overexpressing *PtDGAT2B* (*P* = 0.003 and *P* < 0.01, respectively), where the content of 16:0 and 16:1 in N-limited conditions at 72 h reached 442 nmol mg^−1^ and 443 nmol mg^−1^, respectively, corresponding to a 2- and 1.5-fold increase in comparison to the WT. An increase in the levels of C16 under N-limitation has been reported previously [28, 31] and may be due to the substrate specificity of *DGAT2* genes responding to N deprivation. In this study, we demonstrate that the protein encoded by *PtDGAT2B* has preference for C16 acyl groups. Overall, the accumulation of LC-PUFA (EPA, DPA and DHA) was largely unaffected either by N-depletion or time. However, in all experimental conditions, transgenic cells accumulated higher levels of DHA than WT, with most efficient accumulation observed in DGATElo. Interestingly, after 72 h in N-depleted medium, DGATElo cells accumulated 2.8-fold higher levels of EPA and twofold higher levels of DPA than the single Pt_OtElo5 strain (73 nmol mg^−1^ and 26 nmol mg^−1^, respectively). These results indicate that *PtDGAT2B* plays an important role in FA accumulation under N-replete and N-deplete conditions. Although *PtDGAT2B* has a strong substrate preference for C16 FAs, it also demonstrates the broad substrate specificity of this acyltransferase, particularly towards LC-PUFAs, such as EPA, DPA and DHA.

### Lipidomic analysis of WT and transgenic cells under N-replete and N-deplete conditions

**Fig. 5:**
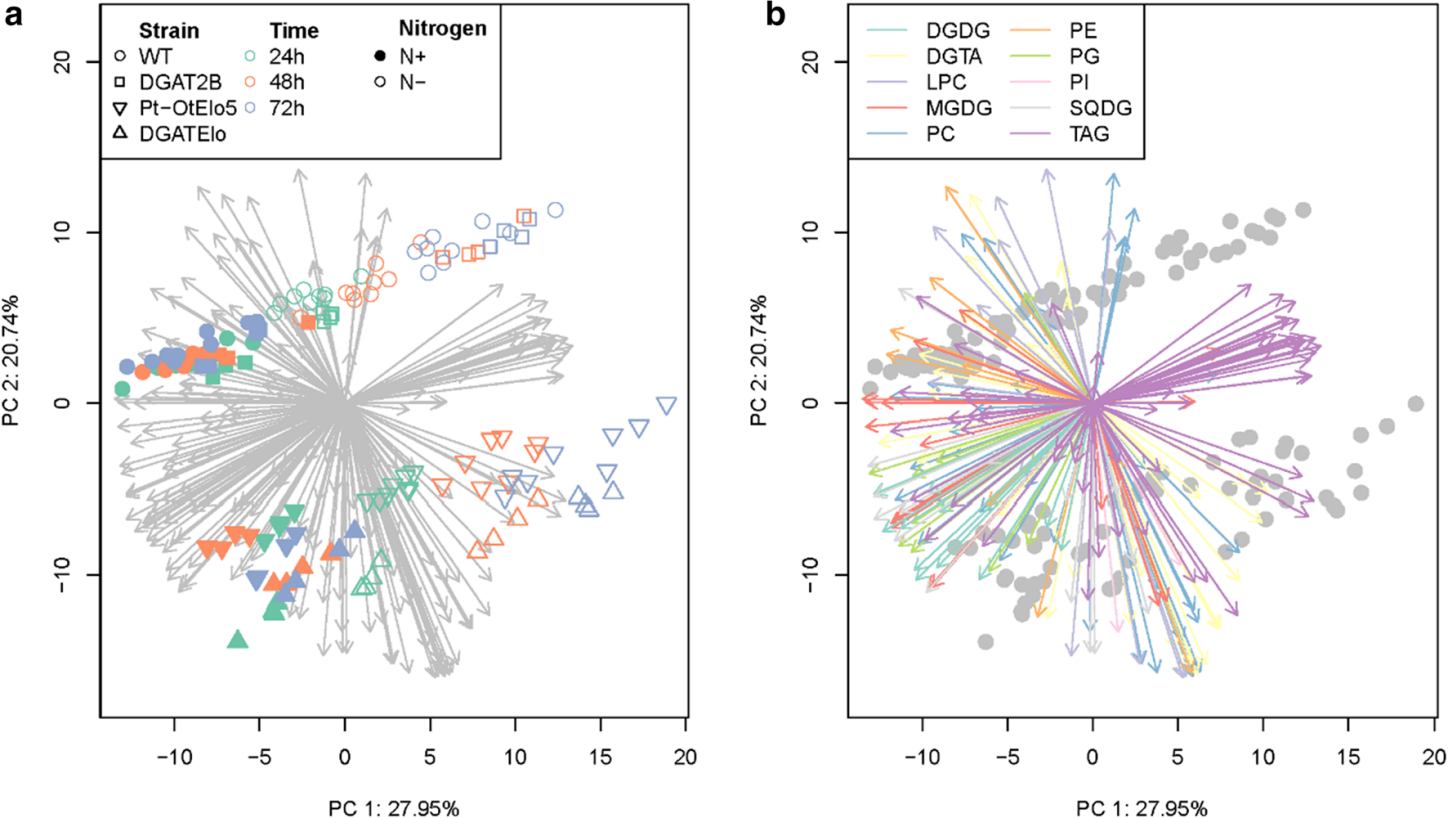
PCA plots of targeted lipidomic analysis (ESI-MS/MS) of *P. tricornutum* strains. Variate biplots of the first two principal axes derived from a principal component analysis characterising the response of cellular lipids over time of *P. tricornutum* (WT, Pt_OtElo5, DGAT2B & DGATElo) lines grown in N-replete and N-deplete conditions of the log transformed data are shown. In both panels, points represent the treatment variates and are coloured in either by (**a**) treatment combinations (strain, time in hours and nitrogen treatment) or (**b**) lipid group (DGDG, MGDG, SQDG, PC, DGTA, LPC, PE, PI, PG, and TAG). The arrows represent the loadings associated with each principal vector

To gain insight into the ability of *PtDGAT2B* to enhance TAG accumulation, we performed a comprehensive analysis comparing glycerolipid profiles at different time points for WT and transgenic strains grown in N-replete and N-deprived conditions. Mass spectrometry (ESI-MS/MS)-based lipidomic approaches have been used previously in our laboratory to characterise *P. tricornutum* lipid turnover and remodelling. The reliability of the analysis was demonstrated by the principle component analysis (PCA), which showed clear clustering of the strain and treatment replicates (Fig. 5). Cells were harvested for analysis at 24, 48 and 72 h, corresponding to E and S phases of growth. Furthermore, the sampling period captured the impact of transgene expression and the application of nitrogen stress, which induces a significant reorganisation of cellular lipid metabolism. A multivariate statistical approach (PCA) was used to decrypt the significant differences observed in such a comprehensive analysis. The first two principal components account for 48.69% of variation within the data and are shown in Fig. 5. Clear patterns are visible in this two-dimensional representation of the data, with the first principal component separating out N− and N+ treatments and within the N− treatments distinguishing between the different time effects. There is separation of the dataset over time under N-deplete conditions, but not N-replete, demonstrating the accumulation and remodelling of lipids in response to N deprivation. The second principal component shows separation of the different transgenic lines, with Pt_OtElo5 and DGATElo clustering together and DGAT2B and WT separating into a second group.

**Fig. 6:**
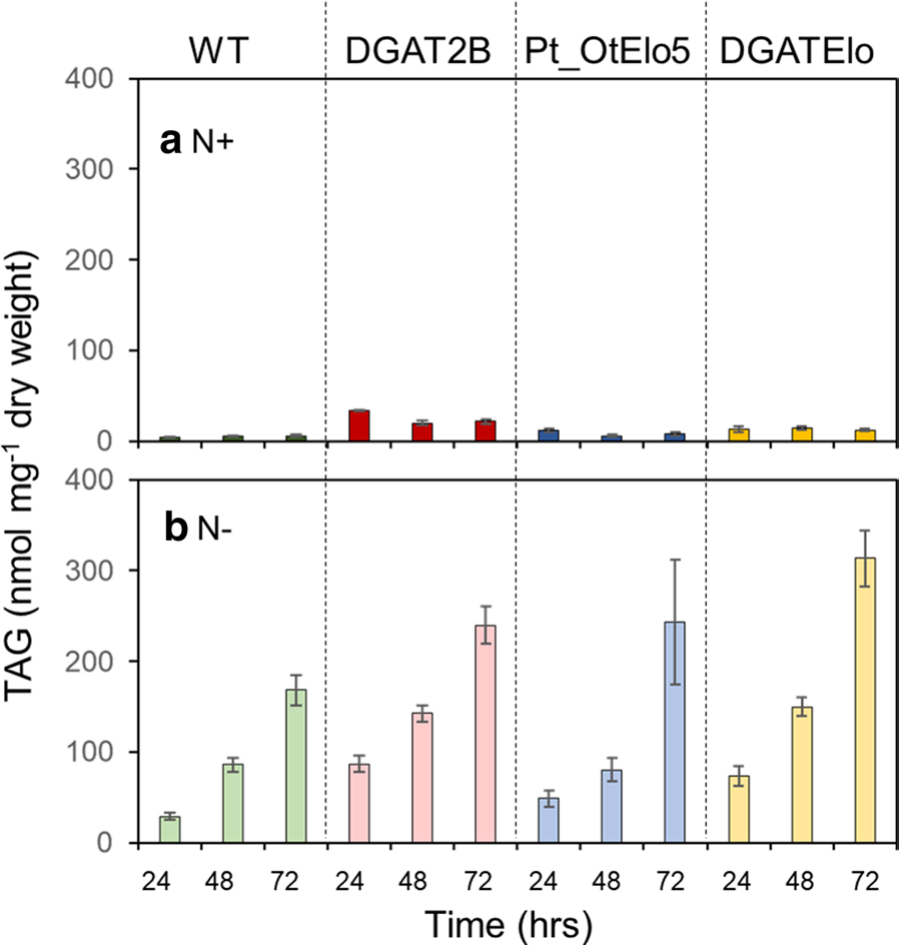
Quantitative analysis of TAG accumulation in *P. tricornutum* cells over a 72 h time course. WT and transgenic (Pt_OtElo5, DGAT2B and DGATElo) *P. tricornutum* lines were grown in (**a**) N-replete (N+) or (**b**) N-deplete (N−) conditions. Total TAG content was determined by ESI-MS/MS analysis. Each measurement is the average of minimum four technical replicates. Error bars indicate standard error

The relative contribution each lipid has on the direction of each principle component was determined. Additional file 5: Table S4 lists the 15 lipids with highest loadings associated with these dimensions. Under N-deplete conditions, the first principal component, a high TAG and low polar lipid response is clear. Specific TAG species with high abundance are 48:2 and 48:3 presumptively 16:0, 16:1-containing TAGs and the EPA-containing TAG 50:3. Other lipid species contributing to this dimension are the galactolipids mono- and digalactosyldiacylglycerol (MGDG and DGDG). The second principal component can be interpreted as an average of selected lipids, namely phosphatidylcholines (PC), Lyso-PCs (LPC) and diacylglycerylhydroxymethyltrimethyl-β-alanine (DGTA), thus high values of principle component two (associated with WT and DGAT strains) correspond to high abundance in these lipids. Compared to WT and DGAT2B, cells expressing the *OtElo5* gene contain lipids (LPC, PC and DGTA) abundant in DPA and DHA. Several important observations can be made from the PCA analysis. When considering the full set of lipid species, cells overexpressing the *PtDGAT2B* gene cluster with WT although the lipid content is altered (Fig. 6, Additional file 4: Figure S1 and Additional File 6: Figure S2). Introduction of the *OtElo5* gene separates out cell lines expressing it under N-replete and N-deplete conditions. Cells expressing the *OtElo5* gene and overexpressing *PtDGAT2B*, individually or in combination, cluster together. These differences between the cell lines are driven by lipids containing DPA and DHA, e.g. DGTA 44:12 amongst others. As expected, the PCA plots show that the biggest impact on distribution is N stress and that this is driven by changes in abundance of TAG. Other lipid species contribute to changes in lipid profile, but they are largely minor components. These data suggest that in response to N stress, de novo biosynthesis of TAG primarily utilising the ER-localised Kennedy pathway, rather than remodelling of membrane lipids, is the major driver in TAG accumulation. The importance of the Kennedy pathway in TAG biosynthesis in stress-induced diatoms has been reported before [2, 3]. This study gives a clear indication of the involvement of one of the major contributors to this pathway, *PtDGAT2B*, in TAG biosynthesis under N-replete conditions.

### Impact of transgene expression on TAG accumulation under N-replete and N-deplete conditions

First, we examined the impact of *PtDGAT2B* overexpression on lipid accumulation in transgenic cells. Both the DGAT2B and DGATElo cells contained elevated TAG compared to WT and Pt_OtElo5 cells irrespective of N conditions at all-time points (Fig. 6 and Additional file 7: Table S5). The results demonstrated that N deprivation is not required for enhanced TAG accumulation in the *PtDGAT2B* overexpressing strains. Under N-replete conditions at 72 h the DGAT2B cells accumulated 22 nmol mg^−1^ DW of TAG, which corresponded to a 3.5-fold increase in comparison to the WT. These results indicated that *PtDGAT2B* overexpression markedly improved lipid productivity in cells of *P. tricornutum.* Since N deprivation stimulates lipid production in various microalgae species, *P. tricornutum* WT and transgenic lines were grown and analysed in N-replete and N-deplete conditions. N depletion induced TAG accumulation in all cell types and resulted in substantially higher TAG than under replete conditions. We observed a ~ twofold increase in TAGs in comparison to the control in the DGATElo cells (with the highest level of 313.7 nmol mg^−1^) and a 1.4-fold increase in DGAT2B and Pt_Elo5 cells (240 nmol mg^−1^ and 243 nmol mg^−1^, respectively) after 72 h.

**Fig. 7:**
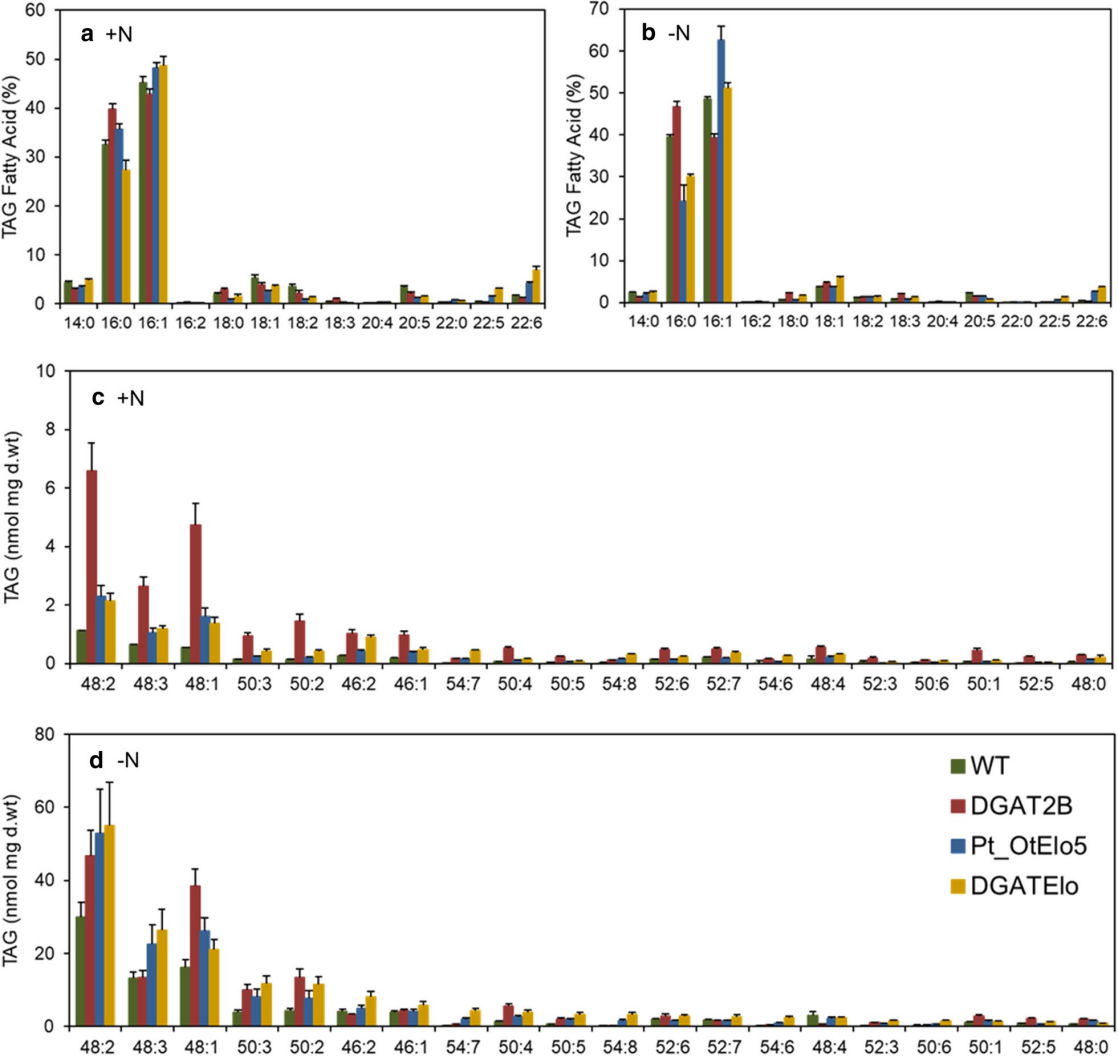
Compositional analysis of fatty acids and molecular species in TAG for *P. tricornutum* cells. Lipids were analysed after 72 h of cultivation in N-replete (+N) and N-deplete (−N) medium. **a**, **b** TAG fatty acid composition; **c**, **d** TAG molecular species composition. Each measurement represents the average of at least four technical replicates. Error bars indicate standard error. Asterisks indicate dominant TAG species, which are significantly different between strains and those which are significantly altered by N stress. Black asterisks indicate TAG species containing 16:0 and 16:1 fatty acids; green asterisks indicate TAG species containing C16–C18 fatty acids; and blue asterisks indicate TAG species containing 20:5, 22:5 and 22:6

To further examine lipid production in *P. tricornutum* cells, TAG accumulation in lipid bodies of the WT and transgenic cells was assessed qualitatively by BODIPY 505/515 staining and confocal microscopy (Additional file 6: Figure S2). Under N-replete conditions oil bodies in WT and Pt_OtElo5 cells remain almost unchanged over the time course, whereas *PtDGAT2B* overexpressing cells contained larger oil bodies at each time point. Neutral lipid content in all cell types increased considerably in N-starved cultures compared to N-replete conditions. DGAT2B and DGATElo cells contained larger and more numerous oil bodies relatively to WT and Pt_OtElo5 over the time. The increase and volume of oil bodies in *P. tricornutum* cells expressing *PtDGAT2B* and/or *OtElo5* were consistent with the increase in neutral lipid content measured by mass spectrometry. To detect the impact of transgene expression on FA assembly in TAG further detailed analysis by ESI-MS/MS was undertaken. FA content in TAG showed that along with the different levels of TAG accumulation between strains, there were also differences in TAG FA profiles and molecular species composition under N-replete and N-deplete conditions. DGATElo contained a significantly higher proportion of DHA in TAG than other strains under both conditions (*P* = 0.0003) (Fig. 7a, b). However, DHA levels were slightly decreased in all cell types under N starvation in agreement with previous observations that LC-PUFAs levels decrease in N-depleted medium [32].

Also, in agreement with previous reports [28, 31] the most abundant TAG species under standard conditions in *P. tricornutum* cells were C16 containing species, 48:1, 48:2 and 48:3. A similar pattern was observed in all transgenic cells. However, the levels of C16 FA-containing species and those consisting of a mix of C16-C18 FA (50:2 and 50:3) in DGAT2B cells increased three- to eightfold compared to WT, thus demonstrating enhanced channelling of C16 FA into TAG in the presence of *PtDGAT2B*. Elevated 54:6, 54:7 and 54:8 corresponding to DHA/DPA-containing TAG species were detected in DGATElo cells (Additional file 8: Figure S3). The levels of TAG species containing C16 FA were dramatically changed with nitrogen treatment. Under N-deplete conditions levels of 48:1, 48:2 and 48:3 TAG were substantially increased in all cell types. DGAT2B and DGATElo cells contained elevated levels of 50:2 and 50:3 TAG. DHA-containing TAG species showed differing responses to N depletion. Notably, DHA TAG 54:7 increased up to 64% in DGATElo, whereas 54:8 and 54:6 increased up to 39 and 8.7%, respectively. Minor new DHA-containing species, e.g. 56:8, 56:9, 56:10 and 56:11, were mainly observed in cells grown in N-deplete media (Additional file 8: Figure S3). The data collectively demonstrated that the broad substrate specificity associated with *PtDGAT2B* facilitated a pathway for DHA synthesis and incorporation into TAG as lipid droplets. Moreover, the combined expression of *PtDGAT2B* and *OtElo5* increases lipid production without altering cellular growth.

### Polar lipid accumulation in WT and transgenic cells under N-replete and N-deplete conditions

To better understand the impact of transgene expression on the lipidome of *DGAT2B* overexpressing cells and determine if the substantial changes in TAG composition under N stress are a result principally of de novo biosynthesis of TAG, as indicated by multivariate analysis, or by lipid remodelling, the polar lipid content of WT and transgenic cells was examined using ESI-MS/MS approaches. The structural reorganisation of chloroplast membranes in response to N depletion and recycling of membrane lipids into TAG has been reported in many microalgae strains. Nitrogen depletion is often accompanied by the movement of LC-PUFA from MGDG and DGDG to TAG [33]. Assessment of the polar lipid classes illustrates a profile that was similar to that of the *P. tricornutum* Pt1 ecotype reported by Abida et al. [28] and dominated by MGDG, PC, phosphatidylglycerol (PG) and DGDG under both N conditions (Additional file 9: Figure S4). The primary impact of N depletion on polar lipids in all strains was a reduction in all classes. Reviewing the individual polar lipid molecular species under N-replete conditions (Additional file 10: Figure S5), the most abundant species in MGDG of all cell types is 36:8, likely comprising of 20:5 and 16:3. Molecular species found in MGDG of DGAT2B cells show similar profiles to that of WT. Transgenic clones expressing the *OtElo5* gene have distinct MGDG profiles from that of WT and DGAT2B strains, and are characterised by enhanced levels of 32:5 and 32:6 (a mix of 16:2 and 16:3) and lower levels of 36:8, correlating with a decrease of EPA due to *OtElo5* activity in these transformants and consequently, reduced import of EPA into the chloroplast via a putative omega pathway [30] (Additional file 10: Figure S5). Under N-deplete conditions there was an overall reduction in EPA-containing 36:6, 36:8, and 36:9 MGDG species in all cell types correlating with a general decrease in EPA content. MGDG 32:5 and 32:6 were also reduced considerably under N depletion. An assessment of other glycolipid species showed differing responses in both DGDG and sulfoquinovosyldiacylglycerol (SQDG) (Additional file 10: Figure S5). Consistent with previous observations [28], 36:7 and 36:6 species were present at high levels in WT and are likely to contain 20:5 and 16:2/16:1. All transgenic clones contain molecular species similar to that of WT indicating that transgene expression did not have a significant impact on DGDG and SQDG. The profile of SQDG in all cells was dominated by 30:1 (14:0/16:1) and 32:1 (16:1/16:0) under N-replete conditions and showed minor decrease under N-deprived conditions. The main EPA-containing species of SQDG, 36.5 (20:5 and 16:0), were present only in WT and DGAT2B cells, correlating with reduced amounts of EPA in *OtElo5*-expressing clones. In contrast to Popko et al. [31], a lower proportion of a betaine glycerolipid has been observed (Additional file 11: Figure S6). This may be due to the different culture growth conditions, e.g. media or light, or analytical methodology used in both experiments.

Under N-replete conditions the dominant phosphatidylcholine (PC) species in WT and DGAT2B were EPA-containing 36:5, 36:6, 38:7 and 40:10 (Additional file 12; Fig. 7a, b). The proportions of these species remained mostly unchanged under both N conditions. The PC pool of *OtElo5* expressing strains in N-replete medium contained highly elevated levels of species incorporating newly synthesised DPA and DHA. The major species in these transformants were those, consisting of the mix of LC-PUFAs and C16 FA: 38:5 (likely 22:5 and 16:0), 38:6 and 38:7 (a mix of 22:6 and C16:0/C16:1), and those with a mix of EPA, DPA and DHA, such as 40:7; 42:9, 42:10 and 42:11. Four new PC molecular species 42:8, 44:10, 44:11 and 44:12 likely containing DHA and DPA were detected in transgenic clones expressing *OtElo5*. An increase in DHA- and DPA-containing species suggests that the synthesis of these LC-PUFAs takes place in the PC pool. Species containing EPA (36:5 and 40:10) in *OtElo5* expressers were markedly reduced, likely corresponding to changes in EPA levels. Interestingly, under N-limited conditions, levels of DHA-containing species, 42:10 and 42:11, increased in DGATElo cells.

Analysis of PG showed a consistent predominance of C16-consisting 32:1 and 32:2 species, and EPA-containing 36:5 and 36:6 species in all the transgenic clones and WT under both N-conditions. Pt_OtElo5 cells contained higher 32:1 in PG compared to WT cells grown in replete and N-deprived medium, and the significance of this is not clear. The measured amount of PG reduced in all cell types under N-deplete conditions (Additional file 12; Fig. 7c, d). Notably, phosphatidylinositol (PI) of all cell types comprised mainly 16:1/16:0 and was stable with N treatment, indicating that this lipid pool was not remodelled in response to N deprivation (Additional file 12; Fig. 7e, f). Phosphatidylethanolamine (PE) profiles were very different between strains grown under N-replete and N-deplete conditions. In N-replete medium PE of WT and DGAT2B cells contained mainly 40:10 (20:5 and 20:5) and 42:11 (20:5 and 22:6) molecular species. New 42:10 (20:5 and 22:5) and enhanced levels of 42:11 species were detected in Pt_OtElo5 and DGATElo transgenic cells (Additional file 12; Fig. 7g, h). The presence of these new species can be attributed to expression of the *OtElo5* gene as they are absent in WT and DGAT2B cells. Jointly, the data for PI and PE indicate that they do not contribute significantly to the biosynthesis of TAG in response to N deprivation.

**Fig. 8:**
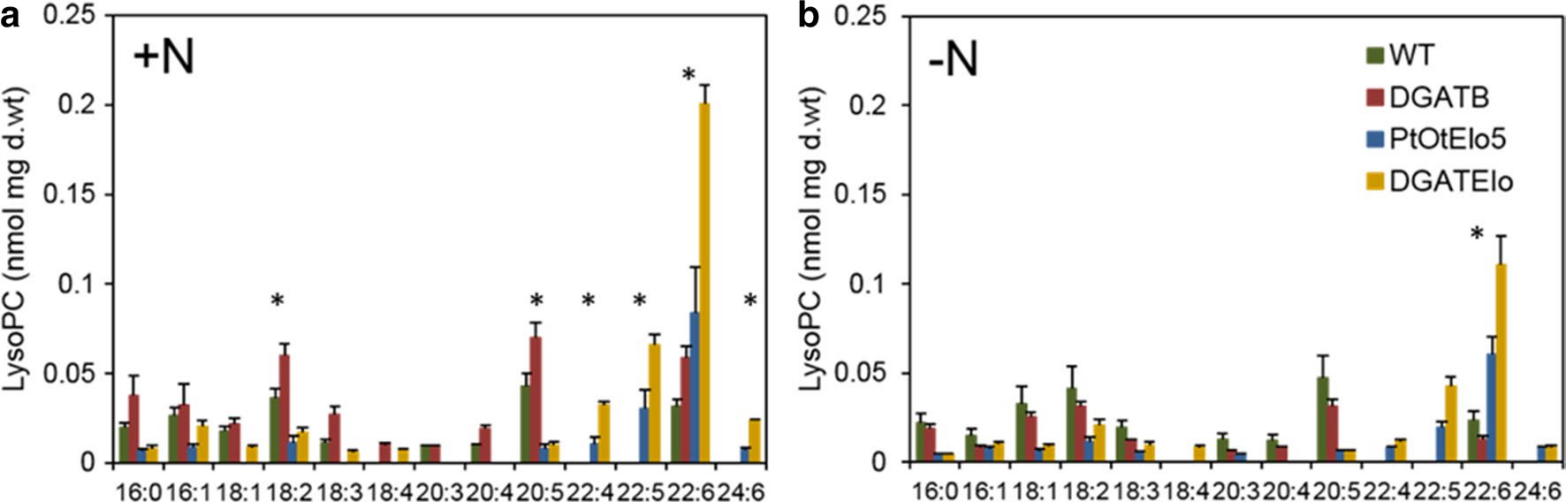
Targeted ESI-MS/MS analysis of Lyso-PC in WT and transgenic *P. tricornutum.* Lipids were analysed in WT, Pt_OtElo5, DGAT2B and DGATElo cells grown in N-replete (+N) and N-deplete (−N) medium at 72 h. Each measurement represents the average of at least four technical replicates. Error bars indicate standard error. FA species that are significantly different to WT, new species or increased under N stress are denoted by an asterisk (*)

To complete our analysis, ESI-MS/MS profiling was used to determine the FA composition of lyso-PC in WT and transgenic cells under the different N treatments (Fig. 8). The major FAs in WT and DGAT2B cells grown under both conditions were 18:2, EPA and DHA, whereas in Pt_OtElo5 and DGATElo transgenic cells FA profiles were dominated by DPA and DHA, the products of Δ5-elongation of EPA and subsequent Δ4-desaturation. In addition, two new FAs, 22:4 (the product of 20:4n-6 Δ5-elongation) and 24:6 (probably the product of 22:6 elongation), were observed in Pt_OtElo5 and DGATElo transformants. There was a twofold increase in DHA-containing lyso-PC in Pt_OtElo5 compared to WT and DGAT2B and a fivefold increase in DGATElo in N-deplete conditions (Fig. 8b). The rise in DHA levels correlated with a reduction of 16:0, 18:1, 18:2, 18:3 and EPA in the *OtElo5* expressing strains. The levels of DHA-containing lyso-PC were twofold higher in DGATElo compared to that of *OtElo5* cells. Overexpression of the *DGAT2B* gene on its own has only small impact on the DHA levels in N-replete cultures. These data indicate a crucial role lyso-PC in LC-PUFA synthesis and incorporation into complex lipid species.

## Discussion

DGATs (acyl-CoA independent Kennedy Pathway) and PDATs (acyl-editing Lands cycle) are the main contributors to oil synthesis in microalgae; of these, DGATs are thought to be the rate-limiting enzymes in TAG accumulation [2]. Most microalgal species, including *P. tricornutum*, have multiple copies of DGAT2 genes. Hence, identification of the best candidates for enhancing LC-PUFA production and TAG accumulation represents a promising approach to the metabolic engineering of strains with desired qualities. However, a functional role for the multiple type 2 DGATs found in algal species remains unclear. In microalgae N stress triggers TAG biosynthesis and often induces DGAT expression [6]. In *P. tricornutum* during N-replete and N-deplete conditions, TAGs and the precursor DAG are mainly composed of 16:0 and 16:1, whereas EPA and DHA are minor species [28, 31]. This suggests that LC-PUFAs are likely transferred to TAGs by either one of the four DGAT2s identified in *P. tricornutum* [13] via the Kennedy pathway or a route involving phosphatidyl diacylglycerol acyltransferase PDAT [2]. We investigated the impact of the overexpression of DGAT2 isoforms in *P. tricornutum* WT and Pt_OtElo5 strains on overall TAG accumulation and specifically DHA partitioning in TAG. Based on the results of the present study, we can suggest that the PDAT-mediated pathway of TAG synthesis does not play a significant role in the response of *P. tricornutum* to nitrogen stress.

FAMEs’ analysis indicated that *PtDGAT2B* can significantly alter FA composition in transgenic clones under N-replete and N-deplete conditions. In clones overexpressing *PtDGAT2B* 16:0 and 16:1 content increased five- and fourfold respectively at 72 h in N-deprived cultures and was considerably higher than that in WT at each time point. Interestingly, DHA levels in strains overexpressing *PtDGAT2B* were higher than that in other strains and not affected by nitrogen depletion. Overexpression of *PtDGAT2B* resulted in significant increases in TAG content in transgenic cells under N-replete and N-deplete conditions at all time points. A fourfold increase in TAG accumulation observed in DGAT2B cells under N-replete conditions demonstrates that the requirement for nutrient depletion to increase lipid accumulation could be overcome using *PtDGAT2B* as a tool for manipulating TAG content. Our observations indicated that transgene expression did not significantly alter growth rate in the different cell lines. This contrasts with a previous study in which a *PtDGAT2D* gene was overexpressed in *P. tricornutum*; cells contained elevated TAG but grew 15% slower than WT [21]. However, any difference in the expression of the *DAGAT2* genes may be due to the differences in growth conditions, e.g. irradiance.

From a biotechnology perspective, the ability to engineer strains that overproduce TAG under normal growth conditions is highly desirable as nutrient depletion negatively affects cell growth. Overexpression of another type 2 DGAT, *NoDGTT5,* in *N. oceanica* CCMP1779 grown in N-replete medium resulted in increased TAG synthesis normally observed only after N deprivation [16]. However, lipid accumulation was accompanied by strongly reduced growth rates normally observed under N-deplete conditions.

N starvation triggers substantial TAG accumulation in all cell types with the highest 37-fold increase in DGATElo cells when compared with N-replete conditions and 1.8-fold higher level of TAG than in the WT in N-deplete medium. Under N stress TAG levels in DGAT2B transformants increased 11-fold when compared with N-replete conditions and ~ 1.5-fold compared with these in WT cells (Fig. 7, Additional file 5: Table S4). Recent studies of DGAT isoforms demonstrated that in *P. tricornutum* only PtDGAT1 was strongly regulated by nitrogen depletion [15], whereas *PtDGAT2B* was highly expressed under N-replete conditions before the onset of TAG accumulation [13]. Our findings suggest that *PtDGAT2B* might contribute to TAG accumulation under both N-replete and N-deplete conditions since we observed significant increase in TAG content in transgenic cells overexpressing *PtDGAT2B* irrespective of N treatment. This is in agreement with the observation by Gong et al. [13] that there are significant differences in the expression patterns between *PtDGAT1* and *PtDGAT2B* genes. *PtDGAT1* was highly responsive to nitrogen limitation implying that *PtDGAT1* gene plays active role in TAG synthesis under N-deplete growth conditions, whereas *PtDGAT2B* was strongly upregulated before the onset of TAG accumulation in N-replete medium, suggesting that *PtDGAT2B* may be directly involved in TAG synthesis under nutrient-replete conditions. We also demonstrated that the *fcp* promoter is suitable for gene overexpression under N deprivation (Fig. 3; Additional file 3: Table S3). This contrasts with the results of expression of an *NoDGTT5* under the control of EF promoter in *N. oceanica* in which elevated TAG was observed under N-replete, but not deplete conditions. However, the use of the native gene promoter did increase TAG biosynthesis under deplete conditions [16].

Lipidomic analysis demonstrated that *PtDGAT2B* possesses broad substrate specificity and can incorporate different acyl-CoA species, including C16 and LC-PUFAs. Overexpression of *DGAT2B* resulted in an increase in TAGs containing C16 species, whereas cells expressing *OtElo5* accumulated elevated levels of LC-PUFA containing TAG (Fig. 7). The levels of DHA-containing TAG species were higher in cells coexpressing both *OtElo5* and *DGAT2B* indicating that *PtDGAT2B* enhances the incorporation and transfer of endogenous C16:0 and C16:1 fatty acids and newly synthesised DPA and DHA into TAG.

Changes in membrane lipids were principally observed in MGDG, DGDG and PG reflecting the rapid senescence of chloroplast membranes, whilst total amounts of PC, DGTA and lyso-PC did not change significantly over the time in all strains. Notably there were significant reductions in EPA- and C16-containing MGDG species. Reductions in C16- and EPA-containing PC and DGTA also correlated with an increase in TAG suggesting that these species are entering TAG biosynthesis, possibly via the dynamic lyso-PC pool (Fig. 8). PDAT with broad substrate specificity have been shown to be a major contributor to TAG biosynthesis in *C. reinhardtii* under N-deplete conditions [34]. Up-regulation of PDAT and enzymes associated with TAG biosynthesis under N stress has also been reported in *P. tricornutum* [35]. However, the pool size of polar lipids is not sufficient to account for the increase in TAG indicating that whilst lipid remodelling occurs under N-deplete conditions, de novo TAG biosynthesis is the major driver behind TAG accumulation. Synthesis of TAG requires a carbon source and the decline in photosynthetic capacity (although not directly measured here, it is inferred by the reduction in chloroplast lipids) with N depletion, indicating that the source of this carbon must be from the remaining photosynthetic capacity and stored carbohydrates. This observation is supported by the multivariate principal component analysis (Fig. 6) and has been reported previously by Abida et al. in the Pt1 ecotype of *P. tricornutum* [28] in contrast to a previous report which proposed betaine lipids as major contributors to TAG formation [31]. An effect that is likely due to differences in growth conditions.

## Conclusions

The overall goal of this work was to evaluate the functional role of *PtDGAT2B* in the synthesis and assembly of TAG. In this study we have successfully demonstrated that *PtDGAT2B* is an important contributor to TAG biosynthesis and a useful tool to produce high accumulating lipid strains for industrial biotechnology. By overexpressing *PtDGATB* we have successfully shown how an engineered transgenic strain of *P. tricornutum* can accumulate elevated TAG levels under N-replete growth conditions. Moreover, the application of a two-gene approach, coexpressing endogenous *DGAT2B* with the heterologous Δ5-elongase from the picoalga, *O. tauri*, produced a significant increase in DHA-containing TAG, confirming the broad substrate specificity of this DGAT. Furthermore, heterologous expression of transgenes did not impact cell growth rates demonstrating the advantage of this approach to engineer improved lipid content and composition. DGATElo cells represent a promising source of DHA that contain enhanced TAG levels without the requirement for N depletion. Although nitrogen stress is usually used to enhance lipid productivity, this study provides evidence that it is possible to increase TAG content without nutrient stress and engineer TAG to contain highly desirable FAs such as DHA and DPA. Previous reports [28, 31, 32] indicated that N-depletion results in increase of saturated (mainly C16:0) and C16:1 fatty acids and reduction in LC-PUFAs in TAGs. Under nitrogen limitation lipid productivity may increase, although biomass production is halted. Our approach allows the maintenance of high biomass accumulation in N-replete medium alongside increased lipid content and favourable fatty acid composition, increasing DHA. To match the level of TAG accumulation under N depletion without affecting the cell growth, further knowledge of regulatory mechanisms of lipid production is required.

## Methods

### Strains and growth conditions

*Phaeodactylum tricornutum* ecotype Pt4 was grown in F/2 media [32], at 20 °C with moderate shaking and constant illumination (60 µmol photons m^−2^ s^−1^). For N stress experiments, replete F/2 media contained enriched phosphate (P) and nitrogen (N) and enriched P with no N in deplete conditions as described by Abida et al. [28]. Cells were grown in enriched F/2 media to the log phase of growth. Cells were then pelleted by centrifugation and used to inoculate 50 ml cultures of replete or depleted media at 2.5 × 10^6^ cells/ml. For each strain *n* = 4–9 depending on the cell type. Cells were counted at time points of 24 h, 48 h and 72 h at which point they were harvested by centrifugation, pellets were washed with 3% ammonium formate, pelleted again, snap frozen in liquid nitrogen and freeze dried.

### Plasmid design and cloning

Plasmids were constructed containing either a single *PtDGAT2B* gene or two-gene cassette with *PtDGAT2B* and *OtElo5* genes. The coding sequences for *PtDGAT2A, PtDGAT2B* and *TpDGAT2* were inserted as *KpnI*-*SacI* fragments into position 1 in pPHOS vector under the control of either *fcpA* or EF2 promoter using a multigene vector described previously [23]. The efficacy of the *fcpA* promoter in N-depleted and N-replete conditions was tested and compared to the *EF2* promoter (Additional file 3: Table S3). The codon-optimised for expression in *P. tricornutum* coding sequence for *O. tauri OtElo5* was inserted as *BamHI*-*XbaI* fragment into position 2 of pPHOS to generate double-gene construct DGATElo. Constructs were transformed into *P. tricornutum* via biolistic according to methods previously described [36]. Positive transformants were selected using the *ble* marker and confirmed by PCR.

### Fatty acid analysis

Total FA content of cells was measured by gas chromatography flame ionisation detection (GC-FID) as described previously [23]. Analysis was carried out using Agilent Chemstation software. Total FAMEs were quantified using 17:0 fatty acid standard (Sigma).

### Nile Red staining of non-polar lipids

Non-polar lipids and oil droplets were measured using Nile Red (Sigma-Aldrich) fluorescence staining (excitation at 485–512 nm and emission at 590–610 nm) using a fluorescent microplate reader (Fluostar Omega, BMG Labtech) as described previously [37]. 200 µl culture containing 2.5 × 10^6^ cells in the exponential phase of growth was stained with 50 µl Nile Red stock (2.5 µg ml^−1^ in 25% DMSO). Blank measurements (cells incubated without Nile Red stain) were subtracted from test samples and total fluorescence was expressed by dividing fluorescence over that of WT cells.

### RNA isolation and qRT-PCR conditions

Total RNA was extracted from 10^8^ cells using an RNeasy Plant mini kit (Qiagen, Germany) according to the manufacturer’s instructions, with minor modifications. Breakage of the algal cells was aided by freezing in liquid nitrogen prior to the addition of the Qiagen kit RLT lysis buffer. The cell mixture was then sonicated for 1 min followed by a incubation on ice for 1 min, and this process was repeated twice. RNase-free DNase (Promega, USA) was used to remove traces of genomic DNA and the RNA was subsequently purified using RNeasy mini kit columns (Qiagen). The concentration of the extracted RNA was determined using a spectrophotometer at 260 nm and 0.5 µg was reverse transcribed into cDNA using the Transcriptor First Strand cDNA synthesis kit (Roche Applied Science, Germany) according to the manufacturer’s instructions. *RPS*, encoding the ribosomal small subunit 30S, was used as the housekeeping gene since the expression level of *RPS* has previously been shown to be stable in different conditions [38].

All remaining genes and the corresponding primer pairs used, designed with the Primer3Plus program, are listed in Additional file 13: Table S6. Relative gene expression levels were analysed using the 2^−∆∆CT^ method using a LightCycler^®^ 96 System (Roche Applied Science). Reactions were started by adding 20 ng cDNA to a mixture containing 1× SYBR Green PCR Master Mix (Roche Applied Science) and 300 nM of the specific primers in a total volume of 10 µl. The thermal-cycling conditions were as follows: 95 °C for 10 min and 40 cycles at 95 °C for 10 s, 60 °C for 15 s and 15 s at 72 °C. Melting curve analysis was performed to check for primer–dimer artefacts. Primer efficiencies were estimated by performing cDNA serial dilutions using the slope calculation supplied with the LightCycler^®^ 96 System Software (Roche Applied Science). For each gene, 3 independent biological replicates and two technical replicates were performed.

### Neutral lipid staining and visualisation by confocal microscopy

Non-polar lipids and oil droplets were visualised using Bodipy staining and confocal microscopy according to Govender et al. [39]. Cells were grown in nitrogen replete or deplete conditions and non-polar lipids and neutral lipids were stained after 24 h, 48 h and 72 h with BODIPY. 0.5 ml cells were pelleted at low speed and re-suspended in artificial sea water (Sigma) containing 1 µg/ml BODIPY (diluted from a stock of 1 mg/ml in DMSO). Cells were incubated for 10 min, pelleted by centrifugation and washed in sea water. Cells were then re-suspended in 15 µl sea water prior to confocal microscopy (Zeiss LSM 780).

### Quantitative lipid analysis

Total lipids were extracted according to Abida et al. with some modifications [28]. 2 mg freeze dried cells were re-suspended in 4 ml propan-2-ol and heated at 80 °C for 10 min. The solvent was transferred to a 50-ml stoppered conical flask. The pellet was then extracted by grinding with a glass homogeniser in 2 ml methanol followed by 8 ml chloroform. Flasks containing solvent extract were flushed with nitrogen gas and stirred for 1 h at room temperature. Samples were filtered through glass wool which was then washed with 3 ml chloroform:methanol (2:1). 5 ml 1% (w/v) NaCl was added to initiate biphase formation and the chloroform phase dried under nitrogen gas. Total lipid extracts were resuspended in chloroform:methanol:ammonium acetate (1:2.2:0.1), filtered (0.45-μm Millex-FH filters, Merck Millipore, Germany), dried under a stream of nitrogen, flushed with nitrogen, and stored at − 80 °C. Quantitative analyses of TAG and polar lipids, which comprise phospholipids (PC, DGTA, PE, PI, PG, LPC) and galactolipids (DGDG, MGDG and SQDG), were carried out using electrospray ionisation tandem triple-quadrupole mass spectrometry (API 4000 QTRAP, SCIEX; ESI-MS/MS). The lipid extracts were infused at 15 μL/min with an autosampler (HTS-xt PAL, CTC-PAL Analytics AG, Switzerland). Data acquisition and acyl group identification of the polar lipids were done as described in Ruiz-Lopez et al. [40]. DGTA analyses were carried out in positive ion mode by scanning for precursors of *m*/*z* 236. The internal standards for polar lipids were supplied by Avanti (Alabaster, AL, USA), incorporated as 0.857 nmol of 13:0-LPC, 0.086 nmol of di24:1-PC, 0.080 nmol of di14:0-PE, 0.800 nmol of di18:0-PI, and 0.080 nmol of di14:0-PG. The standards dissolved in chloroform and 25 μL of the samples in chloroform were combined with chloroform/methanol/300 mM ammonium acetate (300:665:3.5 v/v) to make a final volume of 1 mL. For quantifying TAG, 15 μL of lipid extract and 0.857 nmol of tri15:0-TAG (Nu-Chek Prep, Elysian, MN, USA) were combined with chloroform/methanol/300 mM ammonium acetate (24:24:1.75: v/v), to final a volume of 1 mL for direct infusion into the mass spectrometer. TAG was detected as [M + NH_4_]^+^ ions by a series of different neutral loss scans, targeting losses of fatty acids. The data were processed using the program Lipid View Software (AB-Sciex, Framingham, MA, USA) where isotope corrections are applied. The peak area of each lipid was normalised to the internal standard and further normalised to the weight of the initial sample. There is a variation in ionisation efficiency amongst acyl glycerol species with different fatty acyl groups, and no response factors for individual species were determined in this study; therefore, the values are not directly proportional to the TAG contents of each species. However, the approach does allow a realistic comparison of TAG species across samples in this study.

### Statistical analysis

Principal component analysis (PCA) was done on the complete dataset of 215 lipids in 138 treatment replicates (24 treatment combinations). Data were log transformed after the addition of a small offset calculated as half the minimum non-zero value. PCA was calculated using the correlation matrix, to give equal importance to each variable. The first two principal components account for 48.69% (27.95% and 20.76%) of variation within the data and have clear geometric interpretation. The variation accounted for in the third and fourth roots were 10.17 and 4.93 respectively with little additional interpretation available for these dimensions. Analyses were performed in GenStat 17th Edition and R 3.2.2.

## Supplementary information

**Additional file 1: Table S1.** Selection of independent transgenic lines overexpressing DGAT2 genes during S phase. Fatty acid composition (Mol %) of transgenic clones overexpressing *Pt_DGAT2A*, *Pt_DGAT2B* and *Tp_DGAT2*. Each data point represents one experiment.

**Additional file 2: Table S2**. Selection of independent transgenic lines coexpressing *PtDGAT2B* and *OtElo5*. Fatty acid composition (Mol %) of 10 transgenic clones overexpressing *PtDGAT2B* and OtElo5. Each data point represents one experiment.

**Additional file 3: Table S3.** A comparison of promotor efficacy in response to nitrogen treatment. Fatty acid composition (Mol%) of EPA and DHA in WT and transgenic lines expressing an *O. tauri* Δ5-elongase with either *fcpA* or *EF2* promoter in the E phase of growth.

**Additional file 4: Figure S1.** Fatty acid content (µmol/g) of WT and transgenic *P. tricornutum.* Cells were cultivated in N-replete (N+, darker fill bars) and N-deplete (N-, lighter fill bars) medium at **a** 24, **b** 48 and **c** 72 hours. Each measurement is the average of minimum four technical replicas. Error bars indicate standard deviation.

**Additional file 5: Table S4.** The fifteen lipids with highest loadings associated with the direction of each principal component. The first principal component is a contrast between selected TAGs and galactolipids, e.g. DGDG, meaning high values of principal component one (associated with nitrogen depletion), correspond to low galactolipid abundance, but high TAG response. The second principal component can be interpreted as an average of selected lipids (namely LPCs, PCs and DGTSs), thus high values of principal component two (associated with WT and DGAT strains) correspond to high abundance in these lipids.

**Additional file 6: Figure S2.** Neutral lipid accumulation in WT and transgenic *P. tricornutum* lines. Cells (**a** WT, **b** DGAT2B, **c** Pt_OtElo5 and **d** DGATElo) were grown in replete (N+) and N-deplete conditions. Fluorescence intensity of cells stained with BODYPY 505/515 was measured at 24, 48 and 72 h using confocal microscopy. A 488nm laser was used for the BODIPY stain (yellow) and collected between 493-598 nm, and a 633nm laser for chlorophyll (red) collecting between 647-721 nm.

**Additional file 7: Table S5.** Quantitative ESI-MS/MS analysis of lipid classes. WT and transgenic cells were grown in N-replete and N-deplete conditions and sampled over a 72 h time course. Each measurement is the average of minimum four technical replicates.

**Additional file 8: Figure S3.** DHA-containing TAG. Molecular species as determined by ESI-MS/MS analysis. Lipids were analysed from *P. tricornutum* cells after 72 h of cultivation in N-replete (+N) and N-deplete (−N) medium. Each measurement is the average of minimum four technical replicas. Error bars indicate standard error.

**Additional file 9: Figure S4.** Quantitative lipidomic analysis (ESI-MS/MS) of glycerolipids in WT and transgenic *P. tricornutum* lines. Cells were grown in N-replete (solid fill bars) and N-deplete (no fill bars). Lipids were analysed after 24, 48 and 72 hours of cultivation. Each measurement is the average of minimum four technical replicas. Error bars indicate standard error.

**Additional file 10: Figure S5.** Quantitative analysis of non-phosphorus glycerolipids in *P. tricornutum* WT and transgenic cells. Cultures were grown in N-replete (N+) and N-deplete (N−) medium. Lipids were analysed at 72 h. Each measurement represents the average of at least four technical replicas. Error bars indicate standard error. Abundant lipid species and those significantly different to WT are denoted by asterisks (*). A black asterisk denotes C16-containing species and a red asterisk denotes EPA- and C16-containing species.

**Additional file 11: Figure S6.** Quantitative analysis of diacylglyceryl hydroxymethyltrimethyl-β-alanine (DGTA) in *P. tricornutum* lines. Cells were grown in **a** N-replete (N+) and **b** N-deplete (N−) medium. Each measurement represents the average of at least four technical replicates. Error bars indicate standard error. Abundant lipid species and those significantly different to WT are denoted by asterisks (*). A black asterisk denotes C16-containing species and a red asterisk denotes 20:5- and C16-containing species, and a blue asterisk denotes 22:6 and C16-containing species.

**Additional file 12: Figure S7.** Quantitative analysis of phospholipids in WT and transgenic lines. Cells were grown in N-replete (N+) and N-deplete (N-) medium. Lipids (**a**, **b** PC; **c**, **d** PG; **e**, **f** PI; **g**, **h** PE) were analysed at 72 h. Each measurement is the average of minimum four technical replicates. Error bars indicate standard error. Abundant lipid species, those significantly different to WT and new lipid species are denoted by asterisk (*). Black denotes likely 16:0 containing species (and DPA, panel A); blue denotes a mix of DHA- and C16-containing species; purple denotes a mix of EPA-, DPA- and DHA-containing species; green denotes new DHA-containing species; red denotes EPA-containing species.

**Additional file 13: Table S6.** Primers used in qRT-PCR reactions.

## Data Availability

All data supporting the conclusions of this article are included within the article and in Additional files 1, 2, 3, 4, 5, 6, 7, 8, 9, 10, 11, 12 and 13.

## Abbreviations

DAG: Diacylglycerol
DGAT: Diacylglycerol acyltransferase
DGDG: Digalactosyldiacylglycerol
DGTA: Diacylglycerylhydroxymethyltrimethyl-β-alanine
DGTS: Diacylglyceroltrimethyhomoserine
DHA: Docosahexaenoic acid
GL: Glycolipids
DPA: Docosapentaenoic acid
EPA: Eicosapentaenoic acid
LC-PUFA: Long-chain polyunsaturated fatty acid
MGDG: Monogalactosyldiacylglycerol
NL: Non-polar or neutral lipids
PA: Phosphatidic acid
PC: Phosphatidylcholine
PDAT: Phospholipid diacylglycerol acyltransferase
PE: Phosphatidyethanolamine
PG: Phosphatidylglycerol
PI: Phosphatidylinositol
PL: Phospholipids
PS: Phosphatidylserine
SQDG: Sulfoquinovosyldiacylglycerol
TAG: Triacylglycerol
