# Supplementary material for: Overexpression of an endogenous type 2 diacylglycerol acyltransferase in the marine diatom Phaeodactylum tricornutum enhances lipid production and omega-3 long-chain polyunsaturated fatty acid content
Source: Biotechnol Biofuels. 2020 May 14;13:87. doi: 10.1186/s13068-020-01726-8 (PMC7227059; doi:10.1186/s13068-020-01726-8)
Supplement: Supplementary file 1 — Additional file 1: Table S1. Selection of independent transgenic lines overexpressing DGAT2 genes during S phase. Fatty acid composition (Mol %) of transgenic clones overexpressing Pt_DGAT2A, Pt_DGAT2B and Tp_DGAT2. Each data point represents one experiment. [file 13068_2020_1726_MOESM1_ESM.pdf]

Additional files

Overexpression of an endogenous type 2 diacylglycerol acyltransferase in marine diatom *Phaeodactylum tricornutum* enhances lipid production and omega-3 Long Chain Polyunsaturated Fatty Acid content

Richard P. Haslam[1†], Mary L. Hamilton[1,2†], Chloe Economou[1,3], Richard Smith[1,4], Kirsty Hassall[5], Johnathan A. Napier[1], Olga Sayanova[1*]

[1]Department of Plant Sciences, Rothamsted Research, Harpenden, Herts AL5 2JQ, UK. [2]St Albans Girls School, St Albans, Hertfordshire, AL3 6DB, UK. [3]School of Biological and Chemical Sciences, Queen Mary University of London, Mile End Road, London E1 4NS, UK. [4]Algenuity, Eden Laboratory, Broadmead Road, Stewartby, BEDS MK43 9ND, UK. [5]Department of Computational and Analytical Sciences, Rothamsted Research, Harpenden, Herts AL5 2JQ, UK.

[†]Equal contributor

* Corresponding author – olga.sayanova@rothamsted.ac.uk

Adiitional file 1: Table S1. Selection of independent transgenic lines overexpressing DGAT2 genes during S phase. Fatty acid composition (Mol %) of transgenic clones overexpressing *Pt_DGAT2A*, *Pt_DGAT2B* and *Tp_DGAT2*. Each data point represents one experiment.

Construct	Strain	EPA	DHA
Pt_DGAT2A	1	26.3	1.7
	2	25.7	1.5
	3	23.5	1.5
	4	24.5	1.7
	5	26.2	1.7
WT	1	26.9	1.5
Pt_DGAT2B			
	1	25.8	1.9
	2	19.1	1.6
	3	19.3	1.4
	4	20.7	1.5
	5	21.6	1.9
	6	18.7	1.1
	7	27.3	1.8
	8	19.9	1.3
	9	19.3	1.2
	10	15.6	1.0
WT	1	23.0	1.5
Tp_DGAT2			
	1	30.0	2.0
	2	31.3	2.4
	3	38.4	4.2
	4	31.0	2.3
	5	32.2	2.6
	6	37.0	2.8
	7	33.9	2.1
	8	32.2	2.2
WT	1	33.4	2.2
