# Supplementary material for: Overexpression of an endogenous type 2 diacylglycerol acyltransferase in the marine diatom Phaeodactylum tricornutum enhances lipid production and omega-3 long-chain polyunsaturated fatty acid content
Source: Biotechnol Biofuels. 2020 May 14;13:87. doi: 10.1186/s13068-020-01726-8 (PMC7227059; doi:10.1186/s13068-020-01726-8)
Supplement: Supplementary file 2 — Additional file 2: Table S2. Selection of independent transgenic lines coexpressing PtDGAT2B and OtElo5. Fatty acid composition (Mol %) of 10 transgenic clones overexpressing PtDGAT2B and OtElo5. Each data point represents one experiment. [file 13068_2020_1726_MOESM2_ESM.pdf]

Adiitional file 2: Table S2. Selection of independent transgenic lines coexpressing *PtDGAT2B* and *OtElo5***.** Fatty acid composition (Mol %) of 10 transgenic clones overexpressing *PtDGAT2B* and OtElo5. Each data point represents one experiment.

Construct	DGATElo										WT
Strain	1	2	3	4	5	6	7	8	9	10	1
EPA	25.0	24.5	20.0	14.5	15.0	15.9	16.1	13.9	15.2	24.1	28.0
DPA	0.5	0.7	2.5	2.8	1.2	1.5	1.3	2.6	2.5	0.5	0.0
DHA	2.6	2.7	8.2	9.0	4.8	5.2	5.8	7.9	7.1	3.0	2.3
