# Supplementary material for: Overexpression of an endogenous type 2 diacylglycerol acyltransferase in the marine diatom Phaeodactylum tricornutum enhances lipid production and omega-3 long-chain polyunsaturated fatty acid content
Source: Biotechnol Biofuels. 2020 May 14;13:87. doi: 10.1186/s13068-020-01726-8 (PMC7227059; doi:10.1186/s13068-020-01726-8)
Supplement: Supplementary file 3 — Additional file 3: Table S3. A comparison of promotor efficacy in response to nitrogen treatment. Fatty acid composition (Mol%) of EPA and DHA in WT and transgenic lines expressing an O. tauri Δ5-elongase with either fcpA or EF2 promoter in the E phase of growth. [file 13068_2020_1726_MOESM3_ESM.pdf]

Adiitional file 3: Table S3. A comparison of promotor efficacy in response to nitrogen treatment. Cells were cultuvated in N-replete (N+) and N-deplete (N-) medium. Fatty acid composition (Mol%) of EPA and DHA in WT and transgenic lines expressing an *O. tauri* Δ5-elongase with either *fcpA* or *EF2* promoter in the E phase of growth.

**Sample**	**Repeat**	**EPA (Mol%)**	**DHA (Mol%)**
**WT +N**	1	29.7	3.6
**WT +N**	2	32.5	3.7
**WT +N**	3	35.5	4.7
**fcpA: OtElo5 +N**	1	20.9	11.4
**fcpA: OtElo5 +N**	2	20.7	11.1
**fcpA: OtElo5 +N**	3	19.4	9.7
**EF2: OtElo5 +N**	1	20.5	12.2
**EF2: OtEl05 +N**	2	21.6	12.7
**EF2: OtElo5 +N**	3	20.1	12.8
**WT -N**	1	28.1	3.7
**WT -N**	2	28.7	3.5
**WT -N**	3	26.9	3.4
**fcpA: OtElo5 -N**	1	17.3	14.2
**fcpA: OtElo5-N**	2	16.3	13.4
**fcpA: OtElo5-N**	3	16.9	14.5
**EF2: OtElo5 -N**	1	14.5	14.8
**EF2: OtElo5-N**	2	11.9	11.5
**EF2: OtElo5 -N**	3	13.8	14.1
