# Supplementary material for: Overexpression of an endogenous type 2 diacylglycerol acyltransferase in the marine diatom Phaeodactylum tricornutum enhances lipid production and omega-3 long-chain polyunsaturated fatty acid content
Source: Biotechnol Biofuels. 2020 May 14;13:87. doi: 10.1186/s13068-020-01726-8 (PMC7227059; doi:10.1186/s13068-020-01726-8)

Additional file 4: Figure S1. Fatty acid content (µmol/g) of WT and transgenic *P. tricornutum*. Cells were cultuvated in N-replete (darker fill bars) and N-deplete (N-, lighter fill bars) medium at **a** 24, **b** 48 and **c** 72 hours. Each measurement is the average of minimum four technical replicas. Error bars indicate standard deviation.

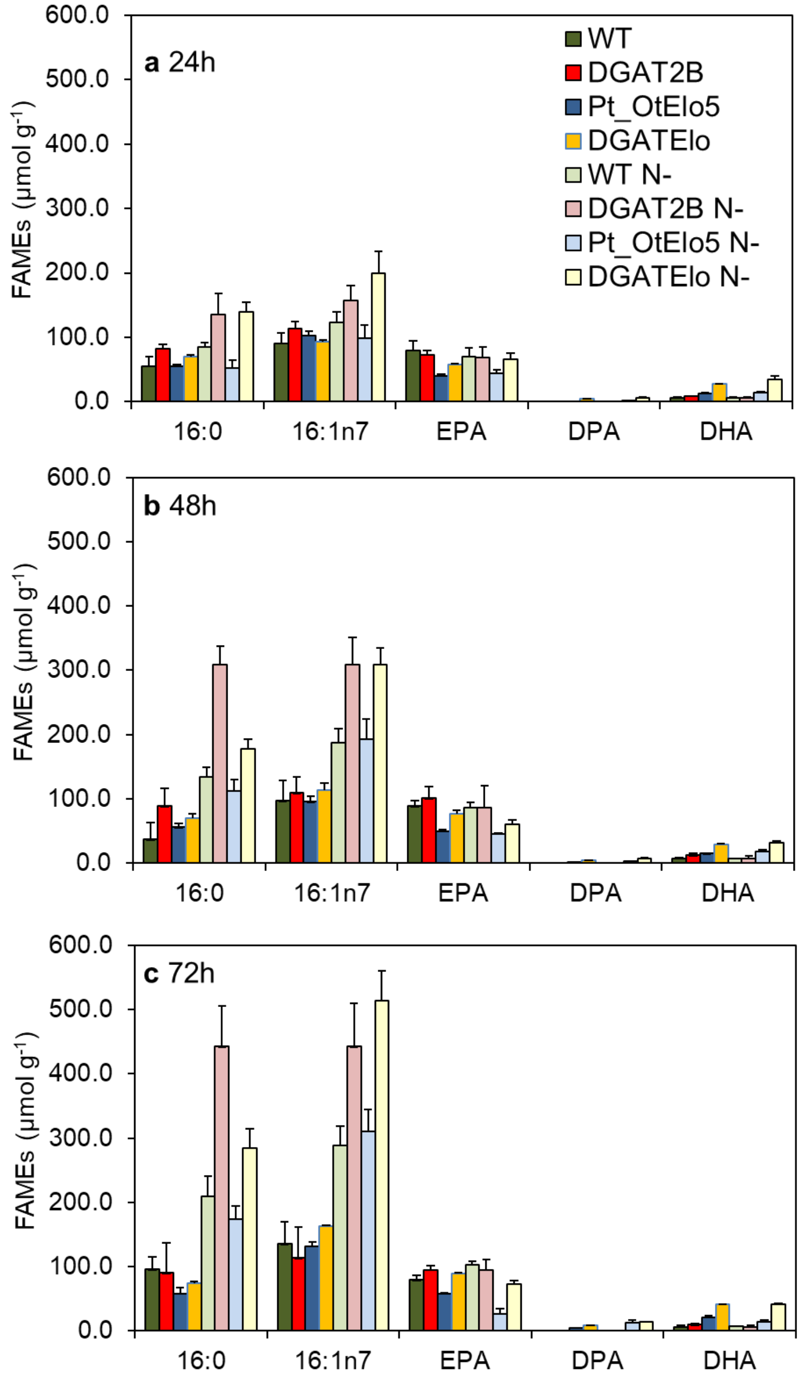

Supplement: Supplementary file 4 — Additional file 4: Figure S1. Fatty acid content (µmol/g) of WT and transgenic P. tricornutum. Cells were cultivated in N-replete (N+, darker fill bars) and N-deplete (N-, lighter fill bars) medium at a 24, b 48 and c 72 hours. Each measurement is the average of minimum four technical replicas. Error bars indicate standard deviation. [file 13068_2020_1726_MOESM4_ESM.pdf]
