# Supplementary material for: Overexpression of an endogenous type 2 diacylglycerol acyltransferase in the marine diatom Phaeodactylum tricornutum enhances lipid production and omega-3 long-chain polyunsaturated fatty acid content
Source: Biotechnol Biofuels. 2020 May 14;13:87. doi: 10.1186/s13068-020-01726-8 (PMC7227059; doi:10.1186/s13068-020-01726-8)
Supplement: Supplementary file 5 — Additional file 5: Table S4. The fifteen lipids with highest loadings associated with the direction of each principal component. The first principal component is a contrast between selected TAGs and galactolipids, e.g. DGDG, meaning high values of principal component one (associated with nitrogen depletion), correspond to low galactolipid abundance, but high TAG response. The second principal component can be interpreted as an average of selected lipids (namely LPCs, PCs and DGTSs), thus high values of principal component two (associated with WT and DGAT strains) correspond to high abundance in these lipids. [file 13068_2020_1726_MOESM5_ESM.pdf]

Adiitional file 5: Table S4. The fifteen lipids with highest loadings associated with the direction of each principal component. The first principal component is a contrast between selected TAGs and galactolipids, *e.g.* DGDG, meaning high values of principal component one (associated with nitrogen depletion), correspond to low galactolipid abundance, but high TAG response. The second principal component can be interpreted as an average of selected lipids (namely LPCs, PCs and DGTSs), thus high values of principle component two (associated with WT and DGAT strains) correspond to high abundance in these lipids.

PC-1			PC-2	
lipid	loading		lipid	loading
MGDG_36_5	-0.1152		LPC_22_4	-0.1365
TAG_48_3	0.1141		LPC_24_6	-0.1361
MGDG_36_7	-0.1136		PC_44_11	-0.1360
MGDG_36_8	-0.1136		DGTA_44_12	-0.1351
SQDG_32_1	-0.1124		PC_44_10	-0.1345
PG_36_5	-0.1119		PE_42_10	-0.1344
TAG_50_3	0.1118		PC_42_10	-0.1341
TAG_48_2	0.1113		PC_42_8	-0.1339
TAG_54_7	0.1105		PC_44_12	-0.1326
SQDG_32_2	-0.1095		DGTA_40_7	-0.1313
DGDG_36_5	-0.1094		DGTA_38_5	-0.1299
TAG_46_2	0.1094		LPC_22_5	-0.1296
SQDG_30_1	-0.1094		PC_42_9	-0.1294
TAG_50_5	0.1087		DGTA_40_8	-0.1278
TAG_54_8	0.1079		LPC_22_6	-0.1255
