# Supplementary material for: Overexpression of an endogenous type 2 diacylglycerol acyltransferase in the marine diatom Phaeodactylum tricornutum enhances lipid production and omega-3 long-chain polyunsaturated fatty acid content
Source: Biotechnol Biofuels. 2020 May 14;13:87. doi: 10.1186/s13068-020-01726-8 (PMC7227059; doi:10.1186/s13068-020-01726-8)

Adiitional file 6: Figure S2. Neutral lipid accumulation in WT and transgenic *P. tricornutum* lines. Cells (**a** WT, **b** DGAT2B, **c** Pt_OtElo5 & **d** DGATElo) were grown in replete (N+) and N-deplete (N-) conditions. Fluorescence intensity of cells stained with BODYPY 505/515 was measured at 24, 48 and 72 h using confocal microscopy. A 488nm laser was used for the BODIPY stain (yellow) and collected between 493-598 nm, and a 633nm laser for chlorophyll (red) collecting between 647-721 nm.

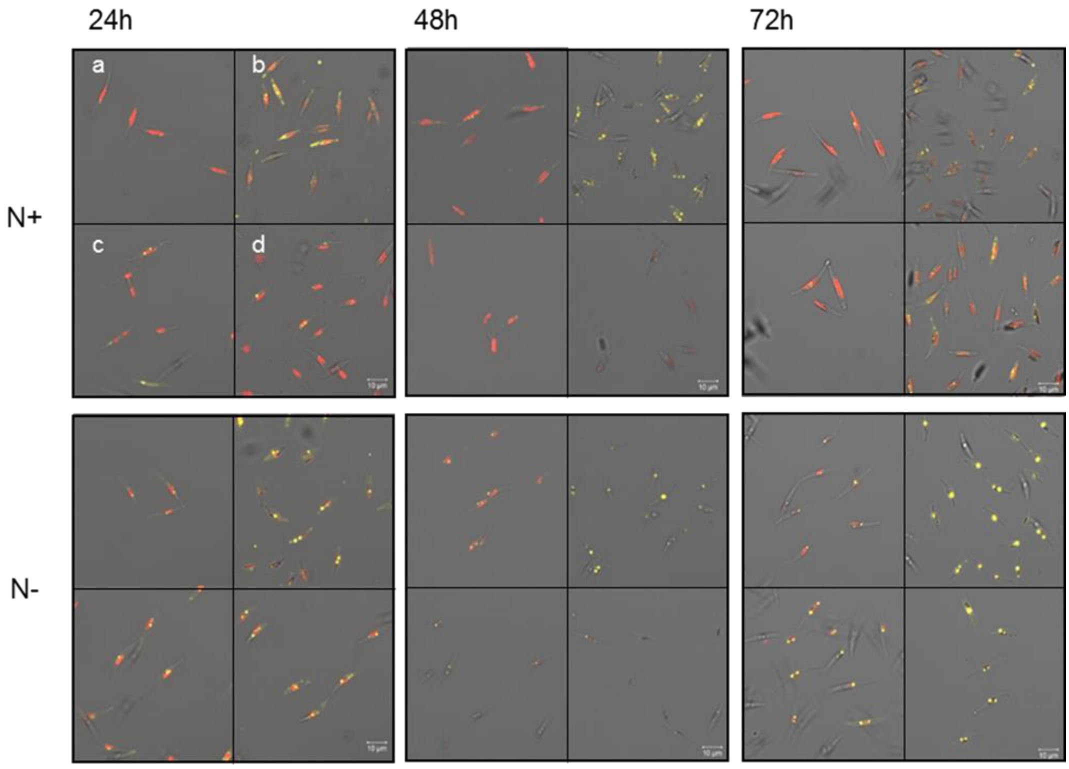

Supplement: Supplementary file 6 — Additional file 6: Figure S2. Neutral lipid accumulation in WT and transgenic P. tricornutum lines. Cells (a WT, b DGAT2B, c Pt_OtElo5 and d DGATElo) were grown in replete (N+) and N-deplete conditions. Fluorescence intensity of cells stained with BODYPY 505/515 was measured at 24, 48 and 72 h using confocal microscopy. A 488nm laser was used for the BODIPY stain (yellow) and collected between 493-598 nm, and a 633nm laser for chlorophyll (red) collecting between 647-721 nm. [file 13068_2020_1726_MOESM6_ESM.pdf]
