# Supplementary material for: Overexpression of an endogenous type 2 diacylglycerol acyltransferase in the marine diatom Phaeodactylum tricornutum enhances lipid production and omega-3 long-chain polyunsaturated fatty acid content
Source: Biotechnol Biofuels. 2020 May 14;13:87. doi: 10.1186/s13068-020-01726-8 (PMC7227059; doi:10.1186/s13068-020-01726-8)
Supplement: Supplementary file 7 — Additional file 7: Table S5. Quantitative ESI-MS/MS analysis of lipid classes. WT and transgenic cells were grown in N-replete and N-deplete conditions and sampled over a 72 h time course. Each measurement is the average of minimum four technical replicates. [file 13068_2020_1726_MOESM7_ESM.pdf]

Additional file 7: Table S5. Quantitative ESI-MS/MS analysis of lipid classes. WT and transgenic cells were grown in N-replete (+N) and N-deplete (-N) conditions and sampled over a 72 h time course. Each measurement is the average of minimum four technical replicates.

Time & Treatment	Strain	Lipid Class (nmol mg d.wt; ±se)									
		PC	DGTA	Lyso-PC	PE	PI	PG	MGDG	DGDG	SQDG	TAG
**24h +N**	**WT**	4.03; 0.69	0.19; 0.04	0.36; 0.07	0.13; 0.01	0.35; 0.02	2.09; 0.30	20.69; 3.21	1.43; 0.26	1.23; 0.17	4.31; 0.68
**24h +N**	**DGAT2B**	6.42; 0.77	0.56; 0.1	0.36; 0.03	0.16; 0.02	0.22; 0.03	1.80; 0.17	21.46; 1.93	1.46; 0.18	1.1; 0.18	33.65; 6.63
**24h +N**	**Pt_OtElo5**	5.07; 0.66	0.19; 0.03	0.39; 0.03	0.13; 0.02	0.94; 0.27	2.21; 0.21	22.03; 1.39	1.49; 0.14	1.13; 0.12	12.70; 1.96
**24h +N**	**EloDGAT**	8.68; 1.06	0.40; 0.05	0.57; 0.03	0.13; 0.01	1.73; 0.21	2.09; 0.37	24.48; 3.88	2.33; 0.68	1.21; 0.1	13.41; 2.91
24h -N	*WT*	3.91; 0.49	0.21; 0.03	0.27; 0.04	0.08; 0.001	0.42; 0.1	0.79; 0.05	14.54; 1.80	0.42; 0.02	0.25; 0.02	29.46; 3.51
24h -N	*DGAT2B*	8.72; 0.73	0.53; 0.08	0.28; 0.01	0.13; 0.03	0.27; 0.04	0.82; 0.02	12.20; 0.88	0.43; 0.01	0.35; 0.07	86.97; 8.75
24h -N	*Pt_OtElo5*	3.88; 0.32	0.21; 0.001	0.30; 0.03	0.09; 0.02	1.07; 0.25	0.84; 0.05	15.32; 1.12	0.52; 0.06	0.48; 0.07	48.85; 8.49
24h -N	*EloDGAT*	9.46; 0.67	0.45; 0.04	0.34; 0.02	0.11; 0.01	1.41; 0.08	1.57; 0.11	15.78; 1.51	0.67; 0.06	0.53; 0.08	74.05; 11.19
**48h +N**	**WT**	4.59; 0.22	0.28; 0.01	0.29; 0.05	0.09; 0.001	0.54; 0.1	2.25; 0.29	22.54; 1.31	1.47; 0.12	1.39; 0.19	5.45; 0.53
**48h +N**	**DGAT2B**	3.46; 0.68	0.31; 0.06	0.26; 0.06	0.08; 0.02	0.12; 0.04	1.94; 0.24	20.84; 3.31	1.50; 0.24	1.18; 0.22	20.05; 2.39
**48h +N**	**Pt_OtElo5**	8.85; 0.48	0.45; 0.02	0.22; 0.02	0.23; 0.01	1.31; 0.26	1.96; 0.15	31.15; 3.42	1.31; 0.15	1.13; 0.12	6.28; 1.17
**48h +N**	**EloDGAT**	6.1; 0.49	0.24; 0.03	0.54; 0.06	0.12; 0.01	1.54; 0.22	2.03; 0.27	16.53; 2.79	1.32; 0.15	1.09; 0.16	14.31; 2.08
48h -N	*WT*	4.93; 0.59	0.28; 0.04	0.22; 0.03	0.09; 0.02	0.68; 0.17	0.72; 0.13	13.06; 1.37	0.58; 0.09	0.23; 0.06	86.21; 7.28
48h -N	*DGAT2B*	2.69; 0.41	0.15; 0.001	0.23; 0.04	0.03; 0.01	0.10; 0.02	0.88; 0.15	5.39; 1.02	0.15; 0.02	0.10; 0.02	142.89; 9.23
48h -N	*Pt_OtElo5*	3.62; 0.52	0.17; 0.02	0.23; 0.04	0.12; 0.03	1.15; 0.08	0.67; 0.07	10.45; 1.83	0.56; 0.06	0.12; 0.01	80.76; 12.98
48h -N	*EloDGAT*	6.17; 0.81	0.28; 0.01	0.27; 0.05	0.08; 0.01	1.32; 0.26	0.83; 0.1	8.57; 1.43	0.56; 0.09	0.12; 0.03	150.3; 10.68
**72h +N**	**WT**	4.82; 0.49	0.28; 0.04	0.21; 0.03	0.13; 0.02	0.49; 0.09	1.71; 0.25	22.94; 3.25	1.32; 0.18	1.21; 0.17	6.27; 0.81
**72h +N**	**DGAT2B**	6.61; 0.95	0.51; 0.13	0.35; 0.05	0.16; 0.03	0.24; 0.04	1.43; 0.29	18.63; 0.70	1.15; 0.4	1.24; 0.17	21.95; 2.49
**72h +N**	**Pt_OtElo5**	7.1; 0.91	0.31; 0.04	0.17; 0.05	0.19; 0.03	0.93; 0.16	1.92; 0.28	23.81; 3.89	1.31; 0.24	1.09; 0.17	8.47; 1.66
**72h +N**	**EloDGAT**	4.03; 0.68	0.27; 0.08	0.40; 0.03	0.089; 0.02	1.04; 0.20	1.35; 0.26	17.01; 3.36	1.26; 0.33	0.99; 0.28	12.75; 0.97
72h -N	*WT*	3.66; 0.41	0.20; 0.03	0.23; 0.06	0.05; 0.01	0.28; 0.06	0.30; 0.06	8.95; 0.91	0.33; 0.05	0.16; 0.03	168.3; 16.55
72h -N	*DGAT2B*	4.74; 0.65	0.27; 0.05	0.15; 0.02	0.04; 0.01	0.15; 0.03	0.17; 0.01	5.55; 0.4	0.20; 0.02	0.17; 0.03	240.19; 20.23
72h -N	*Pt_OtElo5*	4.56; 0.96	0.17; 0.03	0.18; 0.02	0.16; 0.06	0.91; 0.11	0.54; 0.08	5.01; 0.6	0.5; 0.13	0.5; 0.13	243.41; 69.11
72h -N	*EloDGAT*	6.05; 0.3	0.24; 0.01	0.24; 0.03	0.06; 0.001	1.34; 0.14	0.51; 0.03	5.84; 0.18	0.54; 0.02	0.13; 0.01	313.77; 30.81
