# Supplementary material for: Overexpression of an endogenous type 2 diacylglycerol acyltransferase in the marine diatom Phaeodactylum tricornutum enhances lipid production and omega-3 long-chain polyunsaturated fatty acid content
Source: Biotechnol Biofuels. 2020 May 14;13:87. doi: 10.1186/s13068-020-01726-8 (PMC7227059; doi:10.1186/s13068-020-01726-8)

Adiitional file 8: Figure S3. DHA-containing TAG. Molecular species as determined by ESI-MS/MS analysis. Lipids were analysed from *P. tricornutum* cells after 72 hours of cultivation in N-replete (N+) and N-deplete (N-) medium. Each measurement is the average of minimum four technical replicas. Error bars indicate standard error.

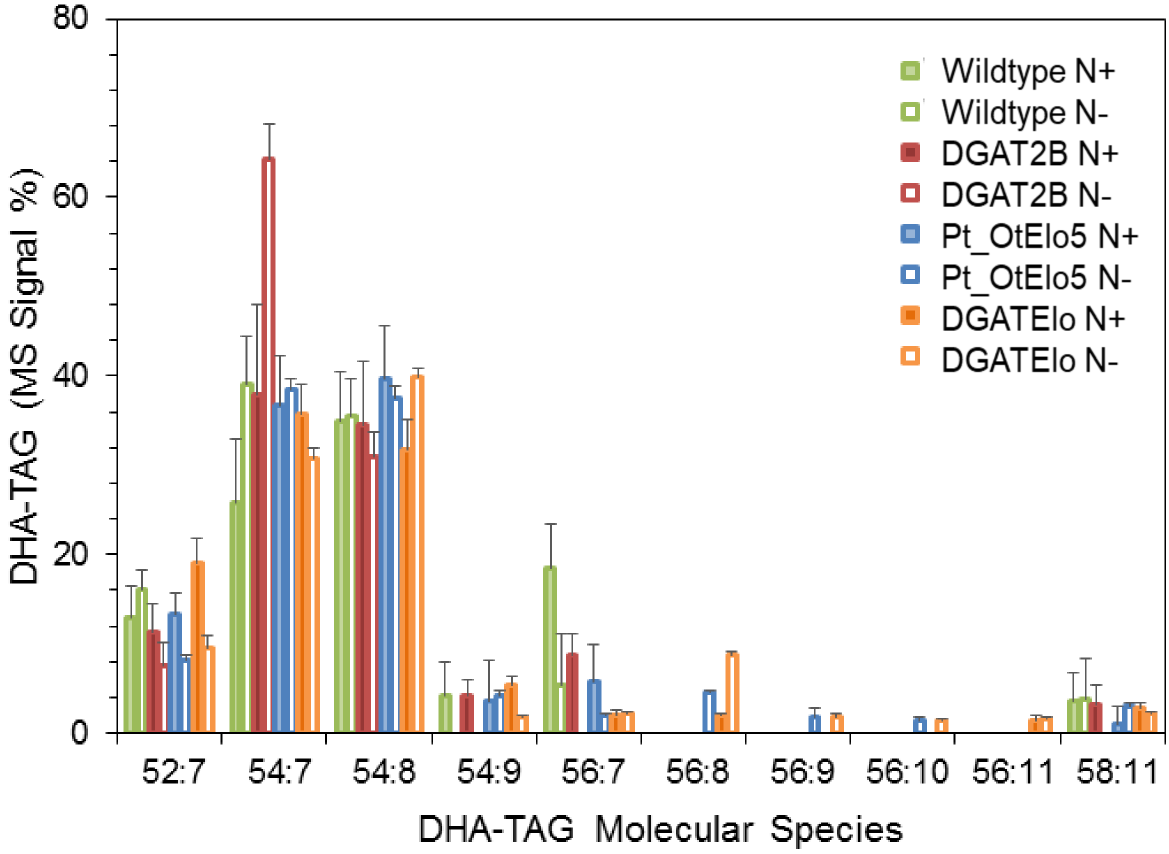

Supplement: Supplementary file 8 — Additional file 8: Figure S3. DHA-containing TAG. Molecular species as determined by ESI-MS/MS analysis. Lipids were analysed from P. tricornutum cells after 72 h of cultivation in N-replete (+N) and N-deplete (−N) medium. Each measurement is the average of minimum four technical replicas. Error bars indicate standard error. [file 13068_2020_1726_MOESM8_ESM.pdf]
