# Supplementary material for: Overexpression of an endogenous type 2 diacylglycerol acyltransferase in the marine diatom Phaeodactylum tricornutum enhances lipid production and omega-3 long-chain polyunsaturated fatty acid content
Source: Biotechnol Biofuels. 2020 May 14;13:87. doi: 10.1186/s13068-020-01726-8 (PMC7227059; doi:10.1186/s13068-020-01726-8)

Adiitional file 9: Figure S4. Quantitative lipidomic analysis (ESI-MS/MS) of glycerolipids in WT and transgenic *P. tricornutum* lines. Cells were grown in N-replete (solid fill bars) and N-deplete (no fill bars). Lipids were analysed after 24, 48 and 72 hours of cultivation. Each measurement is the average of minimum four technical replicas. Error bars indicate standard error.

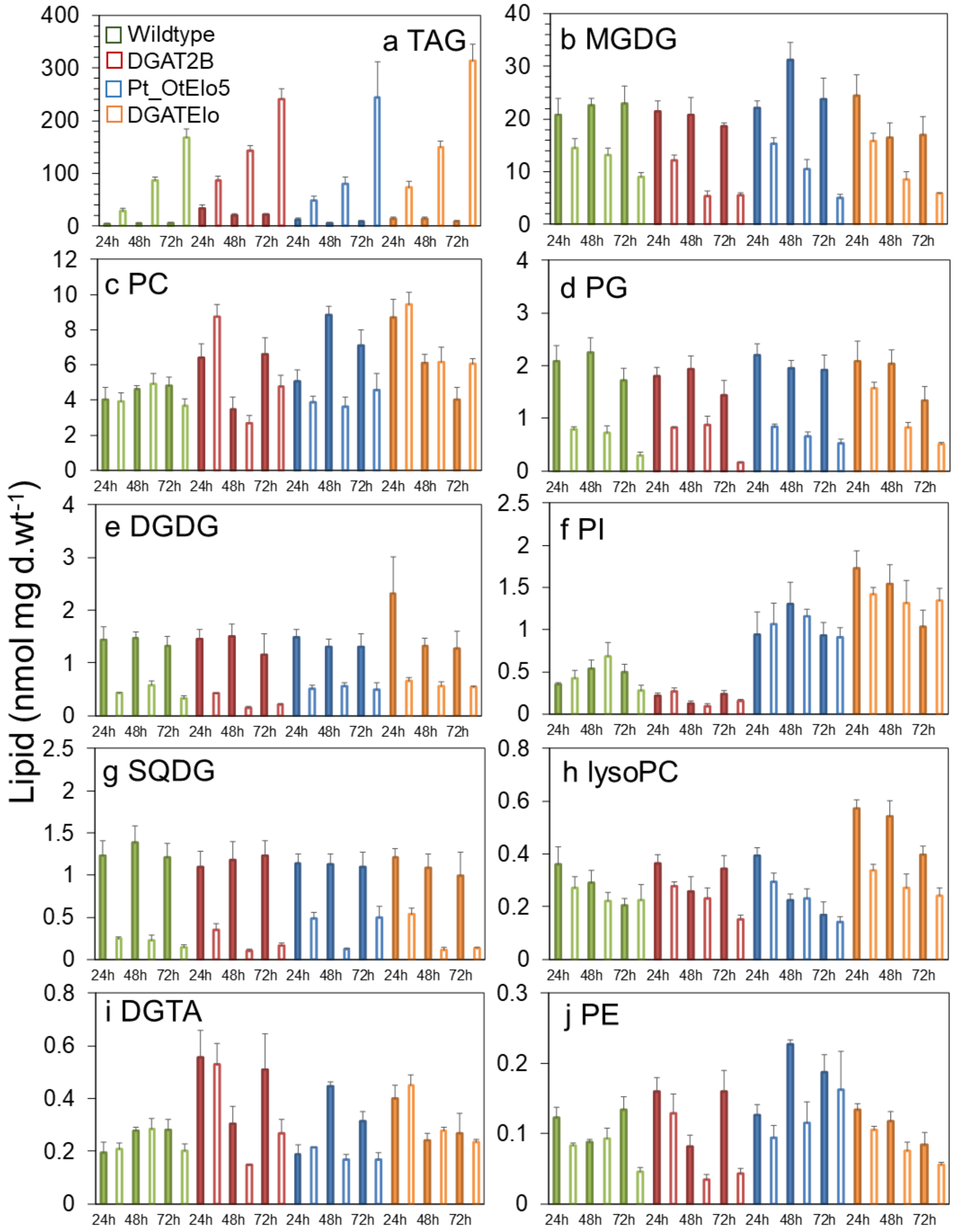

Supplement: Supplementary file 9 — Additional file 9: Figure S4. Quantitative lipidomic analysis (ESI-MS/MS) of glycerolipids in WT and transgenic P. tricornutum lines. Cells were grown in N-replete (solid fill bars) and N-deplete (no fill bars). Lipids were analysed after 24, 48 and 72 hours of cultivation. Each measurement is the average of minimum four technical replicas. Error bars indicate standard error. [file 13068_2020_1726_MOESM9_ESM.pdf]
