# Supplementary material for: Overexpression of an endogenous type 2 diacylglycerol acyltransferase in the marine diatom Phaeodactylum tricornutum enhances lipid production and omega-3 long-chain polyunsaturated fatty acid content
Source: Biotechnol Biofuels. 2020 May 14;13:87. doi: 10.1186/s13068-020-01726-8 (PMC7227059; doi:10.1186/s13068-020-01726-8)

Adiitional file 10: Figure S5. Quantitative analysis of non-phosphorus glycerolipids in *P. tricornutum* WT and transgenic cells. Cultures were grown in N-replete (+N) and N-deplete (-N) medium. Lipids were analysed at 72 h. Each measurement represents the average of at least four technical replicas. Error bars indicate standard error. Abundant lipid species and those significantly different to WT are denoted by asterisks (*). A black asterisk denotes C16-containing species and a red asterisk denotes EPA- and C16-containing species.

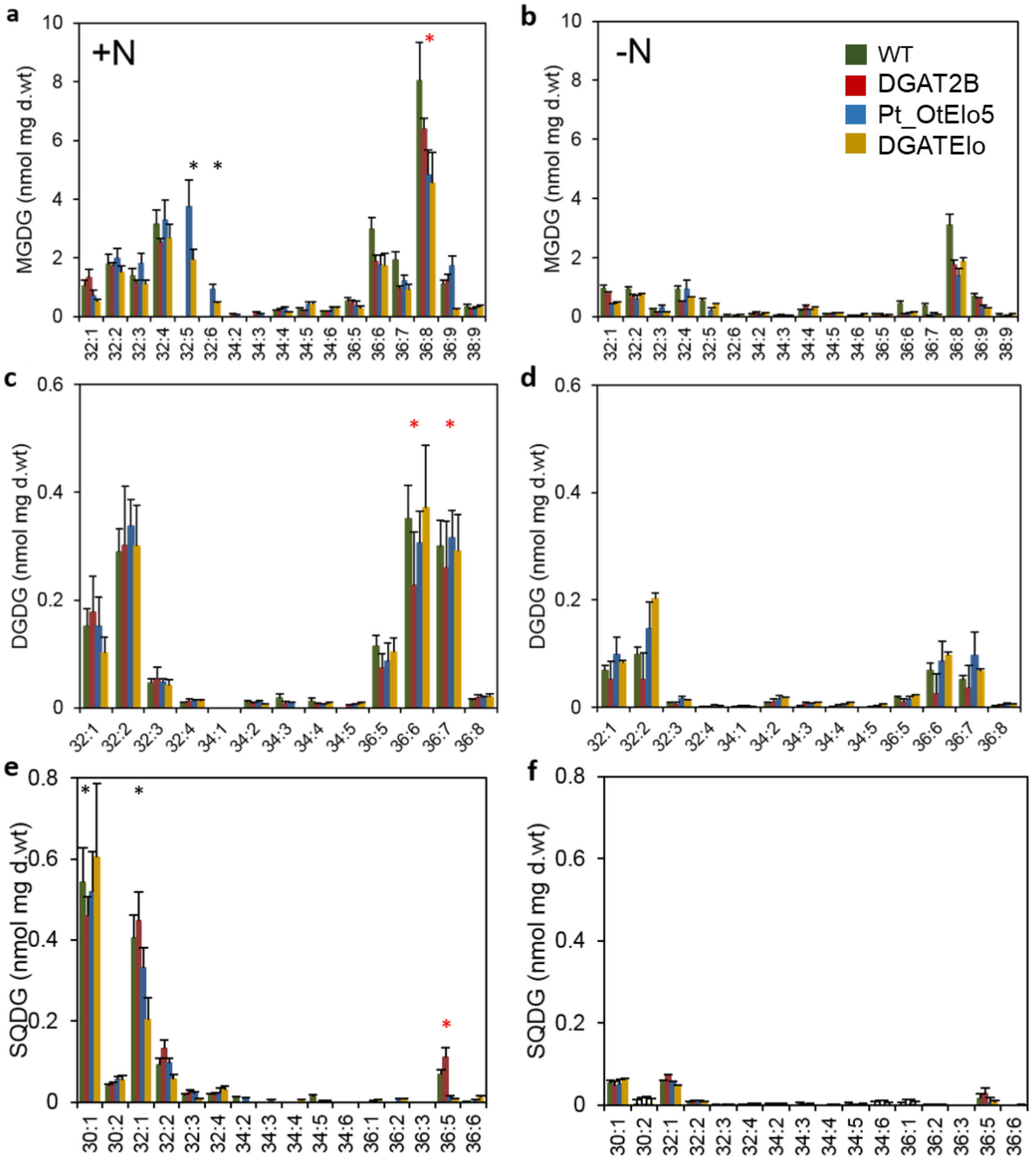

Supplement: Supplementary file 10 — Additional file 10: Figure S5. Quantitative analysis of non-phosphorus glycerolipids in P. tricornutum WT and transgenic cells. Cultures were grown in N-replete (N+) and N-deplete (N−) medium. Lipids were analysed at 72 h. Each measurement represents the average of at least four technical replicas. Error bars indicate standard error. Abundant lipid species and those significantly different to WT are denoted by asterisks (*). A black asterisk denotes C16-containing species and a red asterisk denotes EPA- and C16-containing species. [file 13068_2020_1726_MOESM10_ESM.pdf]
