# Supplementary material for: Overexpression of an endogenous type 2 diacylglycerol acyltransferase in the marine diatom Phaeodactylum tricornutum enhances lipid production and omega-3 long-chain polyunsaturated fatty acid content
Source: Biotechnol Biofuels. 2020 May 14;13:87. doi: 10.1186/s13068-020-01726-8 (PMC7227059; doi:10.1186/s13068-020-01726-8)

Adiitional file 11: Figure S6. Quantitative analysis of diacylglyceryl hydroxymethyltrimethyl-β-alanine (DGTA) in *P. tricornutum* lines. Cells were grown in **a** N-replete (+N) and **b** N-deplete (-N) medium. Each measurement represents the average of at least four technical replicates. Error bars indicate standard error. Abundant lipid species and those significantly different to WT are denoted by asterisks (*). A black asterisk denotes C16-containing species and a red asterisk denotes 20:5- and C16-containing species, and a blue asterisk denotes 22:6 and C16-containing species.

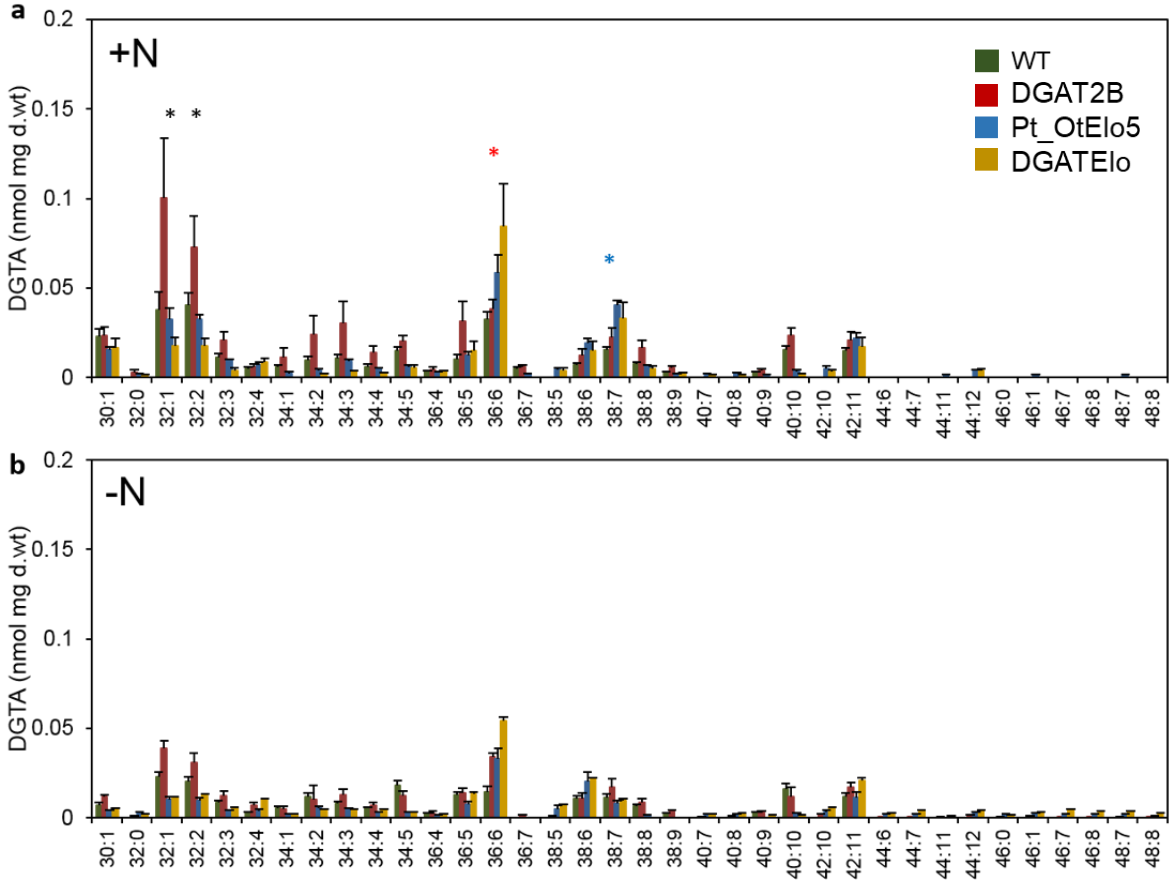

Supplement: Supplementary file 11 — Additional file 11: Figure S6. Quantitative analysis of diacylglyceryl hydroxymethyltrimethyl-β-alanine (DGTA) in P. tricornutum lines. Cells were grown in a N-replete (N+) and b N-deplete (N−) medium. Each measurement represents the average of at least four technical replicates. Error bars indicate standard error. Abundant lipid species and those significantly different to WT are denoted by asterisks (*). A black asterisk denotes C16-containing species and a red asterisk denotes 20:5- and C16-containing species, and a blue asterisk denotes 22:6 and C16-containing species. [file 13068_2020_1726_MOESM11_ESM.pdf]
