# Supplementary material for: Overexpression of an endogenous type 2 diacylglycerol acyltransferase in the marine diatom Phaeodactylum tricornutum enhances lipid production and omega-3 long-chain polyunsaturated fatty acid content
Source: Biotechnol Biofuels. 2020 May 14;13:87. doi: 10.1186/s13068-020-01726-8 (PMC7227059; doi:10.1186/s13068-020-01726-8)

Additional file 12: Figure S7. Quantitative analysis of phospholipids in WT and transgenic lines. Cells were grown in N-replete (+N) and N-deplete (-N) medium. Lipids (**a** & **b**, PC; **c** & **d**, PG; **e** & **f**, PI; **g** & **h**, PE) were analysed at 72 h. Each measurement is the average of minimum four technical replicates. Error bars indicate standard error. Abundant lipid species, those significantly different to WT and new lipid species are denoted by asterisk (*). Black denotes likely 16:0 containing species (and DPA, panel A); blue denotes a mix of DHA- and C16-containing species; purple denotes a mix of EPA-, DPA- and DHA-containing species; green denotes new DHA-containing species; red denotes EPA-containing species.

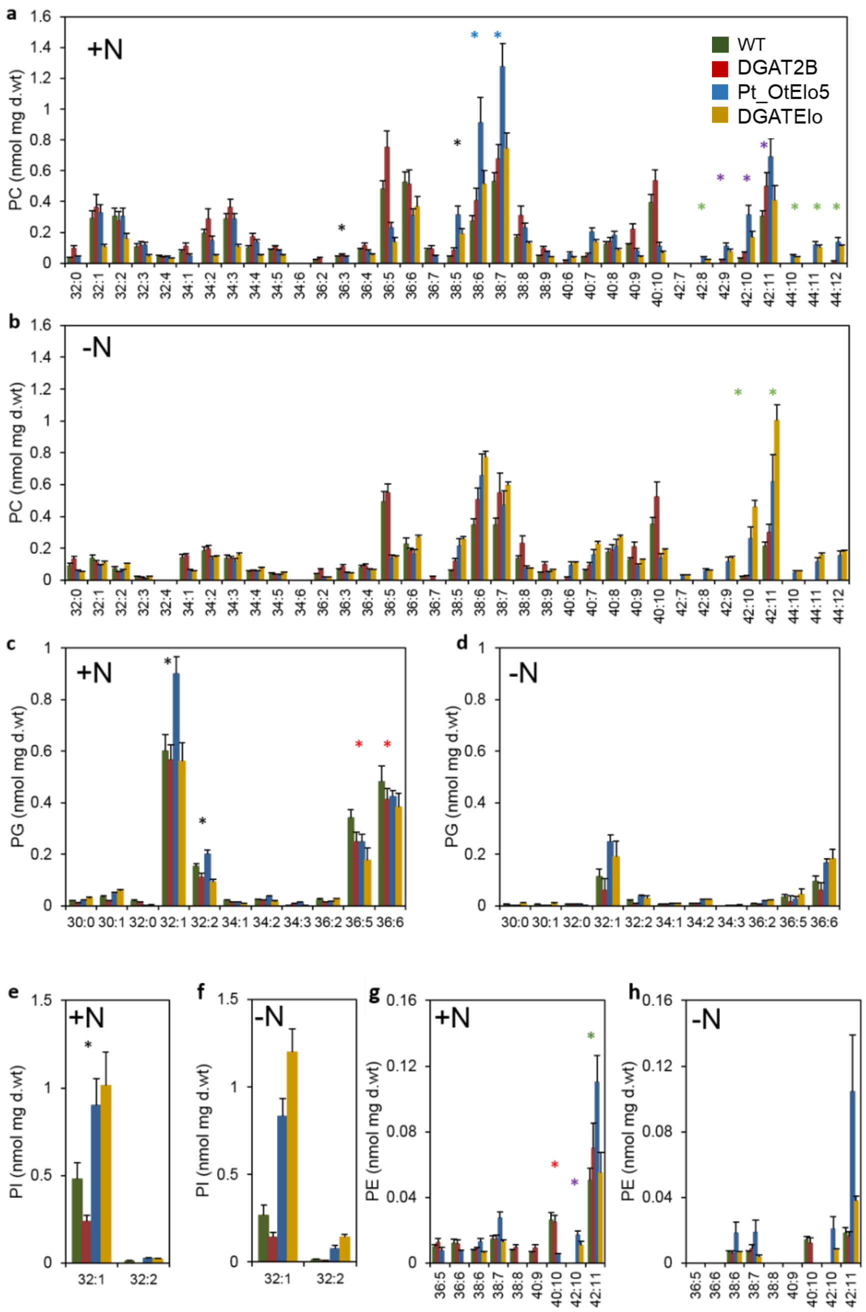

Supplement: Supplementary file 12 — Additional file 12: Figure S7. Quantitative analysis of phospholipids in WT and transgenic lines. Cells were grown in N-replete (N+) and N-deplete (N-) medium. Lipids (a, b PC; c, d PG; e, f PI; g, h PE) were analysed at 72 h. Each measurement is the average of minimum four technical replicates. Error bars indicate standard error. Abundant lipid species, those significantly different to WT and new lipid species are denoted by asterisk (*). Black denotes likely 16:0 containing species (and DPA, panel A); blue denotes a mix of DHA- and C16-containing species; purple denotes a mix of EPA-, DPA- and DHA-containing species; green denotes new DHA-containing species; red denotes EPA-containing species. [file 13068_2020_1726_MOESM12_ESM.pdf]
