# Supplementary material for: Overexpression of an endogenous type 2 diacylglycerol acyltransferase in the marine diatom Phaeodactylum tricornutum enhances lipid production and omega-3 long-chain polyunsaturated fatty acid content
Source: Biotechnol Biofuels. 2020 May 14;13:87. doi: 10.1186/s13068-020-01726-8 (PMC7227059; doi:10.1186/s13068-020-01726-8)
Supplement: Supplementary file 13 — Additional file 13: Table S6. Primers used in qRT-PCR reactions. [file 13068_2020_1726_MOESM13_ESM.pdf]

Additional file 13: Table S6. Primers used in qRT-PCR reactions.

Gene name	Forward (fw)/ reverse (rv) primer	Sequence	Primer pair efficiency
OtELO5	fw	5' GTCACTTGATGGCCACGAAC 3'	2.00
OtELO5	rv	5' GCATTTGAGCCTGGGTGATG 3'	
DGAT2A	fw	5' TCTTCCAAGCGTGGGTAAAG 3'	1.90
DGAT2A	rv	5' TATCGGGAAGCACTCCAAAG 3'	
DGAT2B	fw	5' ACGCGCTGTCGGTACTAAAG 3'	1.92
DGAT2B	rv	5' CCGGAAGCATACAGCAATTT 3'	
TpDGAT	fw	5' ACGAACAAGAAACCCAGACG 3'	1.95
TpDGAT	rv	5' TTCTTGACTCCGAGCCATTC 3'	
